# Polar Lipids Composition, Antioxidant and Anti-Inflammatory Activities of the Atlantic Red Seaweed *Grateloupia turuturu*

**DOI:** 10.3390/md19080414

**Published:** 2021-07-26

**Authors:** Elisabete da Costa, Tânia Melo, Mariana Reis, Pedro Domingues, Ricardo Calado, Maria Helena Abreu, Maria Rosário Domingues

**Affiliations:** 1 Mass Spectrometry Centre, LAQV-REQUIMTE, Department of Chemistry, University of Aveiro, Santiago University Campus, 3810-193 Aveiro, Portugal; taniamelo@ua.pt (T.M.); marianapreis@ua.pt (M.R.); p.domingues@ua.pt (P.D.); mrd@ua.pt (M.R.D.); 2 CESAM-Centre for Environmental and Marine Studies, Department of Chemistry, University of Aveiro, Santiago University Campus, 3810-193 Aveiro, Portugal; 3 ALGAplus—Production and Trading of Seaweed and Derived Products Lda., 3830-196 Ilhavo, Portugal; helena.abreu@algaplus.pt; 4 ECOMARE, CESAM-Centre for Environmental and Marine Studies, Department of Biology, University of Aveiro, Santiago University Campus, 3810-193 Aveiro, Portugal; rjcalado@ua.pt

**Keywords:** ABTS, algae, betaine lipids, COX-2, DPPH, glycolipids, lipidomics, *n*-3 fatty acids, phospholipids, red seaweeds

## Abstract

*Grateloupia turuturu* Yamada, 1941, is a red seaweed widely used for food in Japan and Korea which was recorded on the Atlantic Coast of Europe about twenty years ago. This seaweed presents eicosapentaenoic acid (EPA) and other polyunsaturated fatty acids (PUFAs) in its lipid fraction, a feature that sparked the interest on its potential applications. In seaweeds, PUFAs are mostly esterified to polar lipids, emerging as healthy phytochemicals. However, to date, these biomolecules are still unknown for *G. turuturu*. The present work aimed to identify the polar lipid profile of *G. turuturu*, using modern lipidomics approaches based on high performance liquid chromatography coupled to high resolution mass spectrometry (LC–MS) and gas chromatography coupled to mass spectrometry (GC–MS). The health benefits of polar lipids were identified by health lipid indices and the assessment of antioxidant and anti-inflammatory activities. The polar lipids profile identified from *G. turuturu* included 205 lipid species distributed over glycolipids, phospholipids, betaine lipids and phosphosphingolipids, which featured a high number of lipid species with EPA and PUFAs. The nutritional value of *G. turuturu* has been shown by its protein content, fatty acyl composition and health lipid indices, thus confirming *G. turuturu* as an alternative source of protein and lipids. Some of the lipid species assigned were associated to biological activity, as polar lipid extracts showed antioxidant activity evidenced by free radical scavenging potential for the 2,2′-azino-bis-3-ethyl benzothiazoline-6-sulfonic acid (ABTS^●^^+^) radical (IC_50_ ca. 130.4 μg mL^−1^) and for the 2,2-diphenyl-1-picrylhydrazyl (DPPH^●^) radical (IC_25_ ca. 129.1 μg mL^−1^) and anti-inflammatory activity by inhibition of the COX-2 enzyme (IC_50_ ca. 33 µg mL^−1^). Both antioxidant and anti-inflammatory activities were detected using a low concentration of extracts. This integrative approach contributes to increase the knowledge of *G. turuturu* as a species capable of providing nutrients and bioactive molecules with potential applications in the nutraceutical, pharmaceutical and cosmeceutical industries.

## 1. Introduction

Seaweeds play a central role in oceans food webs, as habitats for marine species that support biodiversity and as bioremediators through nutrient assimilation from seawater, all these features granted them a renewed attention in the blue bioeconomy [1,2,3]. Beyond the context of natural systems, seaweeds have been traditionally used as food in Asian countries and, in recent decades, the use of seaweeds as food has been increasing in the western world [4]. This trend is supported by the growing interest in their nutritional and health benefits promoted by seaweeds in the human diets [4,5]. Moreover, seaweeds are also a sustainable alternative raw material and, in particular, a potential source of polyunsaturated fatty acids (PUFA) and healthy fats to be used in multiple high-end uses [2]. Red seaweeds (Rhodophyta) are a source of many commercially important compounds, such as floridean starch and sulfated galactans, carrageenans and agar, minerals, vitamins, phycobiliproteins and other pigments, mycosporine-like amino acids and unsaturated fatty acids, with multiple applications [6,7]. *Grateloupia turuturu* Yamada, 1941 (also reported in the literature as *G. doryphora*), is a red seaweed native to Japan and Korea where it is traditionally used as food and a source of carrageenan-agar polymers [8]. In recent decades, this seaweed was recorded on the Atlantic Coast of Europe [9], likely originating from aquaculture activities related to oyster farming and/or global shipping [10]. Although *G. turuturu* is non-native species to the Atlantic and may have been erroneously labelled as an invasive species [11], it does not seem to have any negative impacts in coastal environments. In fact, *G. turuturu* seems to contribute to the maintenance of autochthonous seaweed species and to preserve natural biodiversity [12]. 

*Grateloupia* spp. are sea vegetables widely used as a source of proteins and lipids in countries where they are traditionally consumed, being considered a healthy and nutritious food [6]. Information on the composition of *G. turuturu* in the Atlantic Coast is still scarce [6]. The few studies available have shown that it has a high protein content (ca. 14.0%, up to 30.0% DW) [13,14], that it is a good source of essential amino acids [15] and that it has a high carbohydrates content (ca. 41.6%, up to 63.0% DW) [13,14]. The lipid content of *G. turuturu* is low, in line with what is known for other red seaweeds (ca. 0.7%, up to 4.0% DW) [14,16,17]. Nonetheless, lipids of *G. turuturu* are characterized by featuring a high PUFA content [15], as already reported for specimens originating from Brittany [13,16,18] and Portugal (present study). The prevailing PUFA in the lipids of *G. turuturu* is eicosapentaenoic acid (EPA, C20:5*n*-3), considered a fatty acid that is typically supplied by fish, followed by arachidonic acid (AA, C20:4*n*-6) [18,19]. Eicosapentaenoic acid has well-known nutritional and health benefits, including contributing to reduce the synthesis of inflammatory eicosanoids, reactive oxygen species and cytokines and giving rise to anti- inflammatory mediators [20]. In addition, EPA has been associated with the prevention of cardiovascular infections and chronic diseases [21,22] and diabetes [23], as well as exhibiting antimicrobial [23] and antioxidant activities [24]. In seaweeds, FAs are mainly found in polar lipids, such as glycolipids (GL), phospholipids (PL) and betaine lipids, important constituents of cell and plastid membranes [25]. So far, classical chromatographic-based approaches followed by off-line gas chromatography–mass spectrometry (GC–MS) analysis of FAs have been performed to tentatively identify the glycolipids and phospholipids classes on *G. turuturu* [16,19,26], but its polar lipidome at the molecular level has not yet been determined. This characterization is currently achieved by using high-resolution mass spectrometry (MS)-based lipidomic approaches coupled to liquid chromatography (LC), as already described for other red seaweeds [27,28,29,30]. The use of this omics approach makes it possible to identify not only PUFA carriers but also certain molecules with intrinsic bioactive properties.

In the search for bioactive compounds for sustainable use of *Grateloupia* spp., some studies have reported the evaluation of bioactivities of organic solvent extracts of *G. turuturu* (also reported in the literature as *G. doryphora*) and species in the same genus, such as G. *filicina*, *G. elliptica*, *G. imbricata* and *G. lanceola* [7,31,32,33,34]. Methanol, acetone, ethyl acetate and chloroform extracts from *G. filicina* have shown good antioxidants properties as scavengers of reactive oxygen species [35] and capacities of reducing lipid peroxidation in oils and DNA damage in rat lymphocytes [36]. Remarkably, the fat content of some extracts has been found to correlate with antioxidant activity. Ethyl acetate extracts obtained from *G. elliptica* revealed anti-inflammatory activity by showing the capacity to inhibit the production of pro-inflammatory mediators, such as nitric oxide (NO), prostaglandin E2 (PGE2), interleukin-6 (IL-6) and tumor necrosis factor-α (TNF-α) [37]. The biological potential of *Grateloupia* extracts has generated more interest due to their beneficial effects in the prevention of chronic diseases and aging. A supplementation with 20% of *G. turuturu* biomass increased the longevity of *Drosophila melanogaster*, while a formula containing 100% *G. turuturu* achieved the highest antigenotoxic potential against streptonigrin-induced genotoxicity [33]. *Grateloupia turuturu* mixed diet is a promising complementary source of lipids in abalone aquaculture [38], thus reinforcing the potential use of this seaweed as a source of nutritious lipids in feed applications. 

In order to increase our knowledge of this seaweed, in the present study, we evaluated the nutritional value of *G. turuturu* by performing a thorough characterization of the polar lipidome using LC–MS and fatty acid profiling using GC–MS, followed by calculation of health lipid indices. The antioxidant activities of the lipid extracts were carried out by evaluating their potential for scavenging free radicals against the 2,2-diphenyl-1-picrylhydrazyl (DPPH) and 2,2′-azino-bis-3-ethyl benzothiazoline-6-sulfonic acid (ABTS) radicals and the anti-inflammatory activity of the lipid extracts was evaluated based on the capacity to inhibit the cyclooxygenase-2 (COX-2) enzyme. 

## 2. Results

### 2.1. Lipids Content and Carbon/Nitrogen Ratio 

The total lipid content of *G. turuturu* was 0.88% ± 0.25% of dry weight biomass (DW). The total content of lipid, carbon, nitrogen and protein, along with the C/N ratio of *G. turuturu*, are presented in Table 1. The tissue carbon-to-nitrogen ratio (C/N) accounted for 8.30 ± 0.19. The protein content accounted for 26.26% ± 0.69% DW. 

### 2.2. Lipidome of Grateloupia turuturu

**Figure 1:**
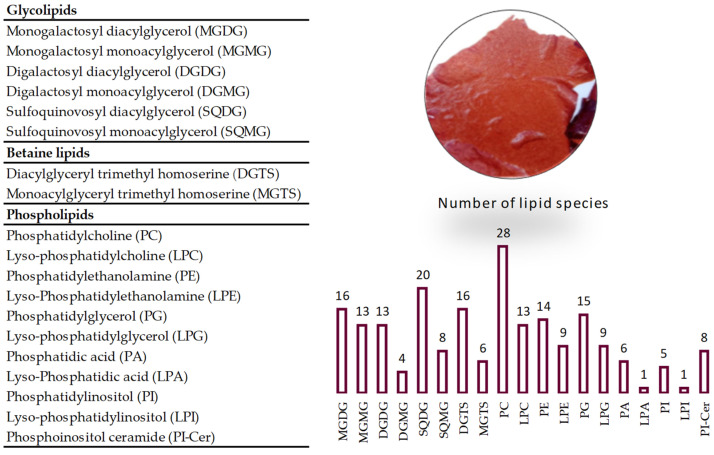
The number of lipid species identified by HILIC–MS, MS/MS in *Grateloupia turuturu* and exact mass measurements with the assignment class of each polar lipids.

A total of 205 lipid species (*m*/*z* values) were identified, with these being distributed over glycolipids (74 species), phospholipids (109 species) and betaine lipids (22 species) (Figure 1). 

#### 2.2.1. Glycolipids Profile of *Grateloupia turuturu*

**Figure 2:**
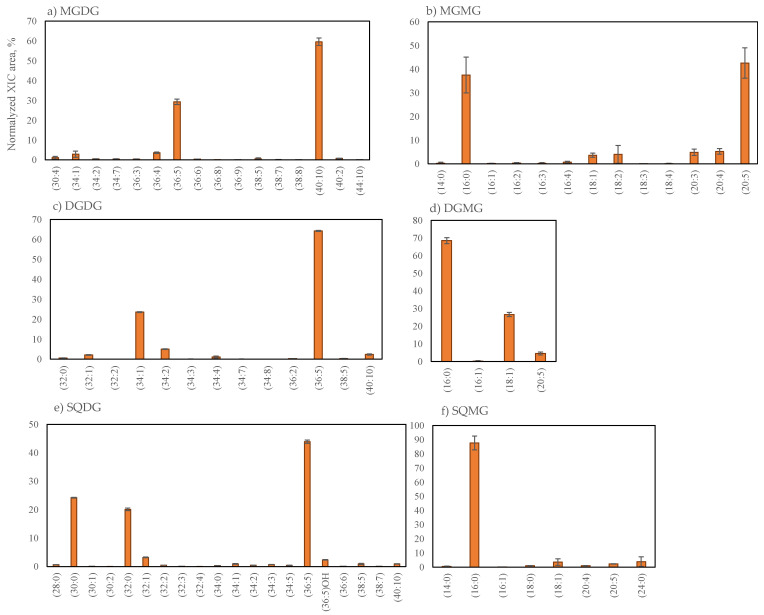
The relative percentage of the normalized peak area of glycolipid species of *Grateloupia turuturu* identified in each class, MGDG (**a**), MGMG (**b**), DGDG (**c**), DGMG (**d**), SQDG (**e**) and SQMG (**f**), calculated as normalized peak area of each lipid species / sum of normalized peak areas for all lipid species of the same class obtained after LC–MS analysis. MGDG (**a**), MGMG (**b**), DGDG (**c**) and DGMG (**d**) were identified in positive mode as [M + NH_4_]^+^ ions and SQDG (**e**) and SQMG (**f**) were identified in negative mode as [M – H]^–^ ions. The number in parentheses (C:N) indicates the number of carbon atoms (C) and the total number of double bounds (N) in the side chains of the fatty acids.

The glycolipids species (GLs) identified in *G. turuturu* included the galactolipid classes MGDG (16 lipid species), DGDG (13 lipid species), MGMG (13 lipid species) and DGMG (4 lipid species) and the classes of the anionic sulfolipid SQDG (20 lipid species) and SQMG (8 lipid species). Galactolipids were identified by LC–MS as [M + NH_4_]^+^ ions and sulfolipids as [M − H]^−^ ions. The identification of the lipids species is provided in Table 2 (Supplementary Materials, Table S1, Figures S2 and S3). The relative percentage of each lipid species within each specific class was calculated and the graphical representation is shown in Figure 2.

Glycolipids species were mainly esterified with C14:0, C16-, C18- and C20-fatty acyl chains and the unsaturation degree of the lipids species (DoU) ranged between 0 and 5 for lyso-GLs (MGMG, DGMG and SQMG), 1 and 10 for MGDG and 0 and 10 for the DGDG and SQDG classes (Table 2). Our data showed that the more abundant lipid species had a total number of 36- and 40-carbons in the case of MGDG, 34- and 40-carbons in DGDG, 30-, 32- and 36-carbons in SQDG, 16- and 20-carbons in MGMG and 16-carbon in DGMG and SQMG (Figure 2). Lipid species bearing C14:0, C16:0, C16:1 and C20:5 were present at up to 10% of relative abundance (RA) in each class. Among the most abundant galactolipids species were MGDG (40:10), assigned as MGDG (20:5/20:5) at *m*/*z* 840.5633, DGDG (36:5) assigned as DGDG (20:5_16:0) at *m*/*z* 956.6315, MGMG (20:5) at *m*/*z* 556.3488, MGMG (16:0) at *m*/*z* 510.3637 and DGMG (16:0) at *m*/*z* 672.4174. In the sulfolipids class are SQDG (36:5) corresponding to SQDG (20:5_16:0) at *m*/*z* 839.4989 and SQMG (16:0) at *m*/*z* 555.2849. Hydroxy-lipid species SQDG (36:5)OH was identified in the SQDG lipid class. 

#### 2.2.2. Betaine Lipids Profile of *Grateloupia turuturu*

**Figure 3:**
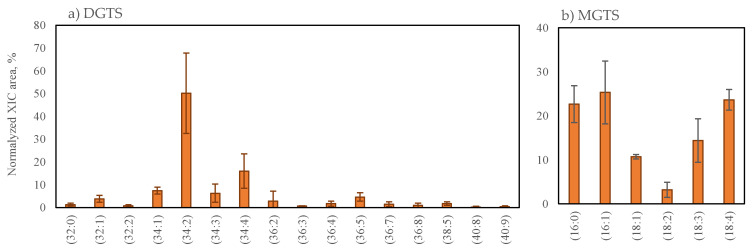
The relative percentage of the normalized peak area of betaine lipid species of *Grateloupia turuturu* identified in each DGTS (**a**) and MGTS (**b**) class, calculated after dividing the normalized peak area of each lipid species/sum of normalized peak areas for all lipid species in the same class obtained after LC–MS analysis. DGTS (**a**) and MGTS (**b**) were identified in positive mode as [M + H]^+^ ions. The number in parentheses (C:N) indicates the number of carbon atoms (C) and double bonds (N) in the side chains of fatty acids.

The betaine lipids diacylglyceryl-N,N,N-trimethyl-homoserine (16 DGTS lipid species) and monoacylglyceryl-N,N,N-trimethyl-homoserine (6 MGTS lipid species) were identified in *G. turuturu*. Lipid species were identified using LC–MS spectra as [M + H]^+^ ions, as described in Table 3. The RA of lipids species within each of the aforementioned classes is shown in Figure 3 and Supplementary Materials, Figure S4. The fragmentation pattern observed in the MS/MS spectra of these lipids is shown in the Supplementary Materials (Table S1, Figure S4b,d). Betaine lipids were mainly esterified into C16- and C18-fatty acyl chains and the unsaturation degree (DoU) of lipids species ranged between 0 and 4 in the case of MGTS, while, in the case of DGTS, the DoU ranged between 0 and 9. The major lipid species of the DGTS class were the 34-carbons DGTS and the most abundant lipid species was DGTS (34:2), assigned to DGTS (16:1_18:1) at *m*/*z* 736.6101. The most abundant MGTS species had 16- and 18-carbons, namely, MGTS (16:1) at *m*/*z* 472.3631, MGTS (16:0) at *m*/*z* 474.3795 and MGTS (18:4) at *m*/*z* 494.3486.

#### 2.2.3. Phospholipids Profile of *Grateloupia turuturu*

**Figure 4:**
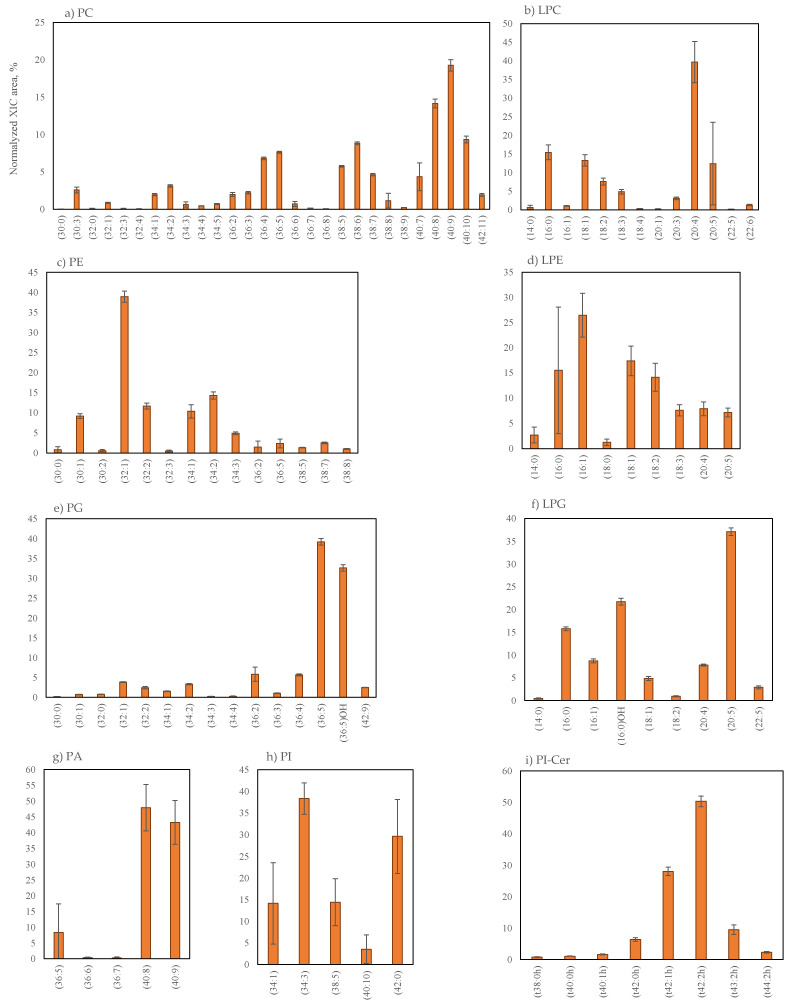
The relative percentage of normalized peak area of the phospholipids lipids classes of *Grateloupia turuturu* identified, PC (**a**), LPC (**b**), PE (**c**), LPE (**d**), PG (**e**), LPG (**f**), PA (**g**), PI (**h**) and PI-Cer (**i**), calculated as normalized peak area of each lipid species / sum of normalized peak areas for all lipid species in the class obtained after LC–MS analysis. PC (**a**) and LPC (**b**) were identified as [M + H]^+^ ions, while PE (**c**), LPE (**d**), PG (**e**), LPG (**f**), PA (**g**), PI (**h**) and PI-Cer (**i**) were identified as [M – H]^−^ ions. The number in parentheses (C:N) indicates the number of carbon atoms (C) and the total number of double bounds (N) in the side chains of fatty acids.

Ten lipid classes were identified in the phospholipids (PLs) of *G. turuturu*: phosphatidylcholine (PC, 28 lipid species) and lyso-PC (LPC, 13 lipid species), phosphatidylethanolamine (PE, 14 lipid species) and lyso-PE (LPE, 9 lipid species), phosphatidylglycerol (PG, 15 lipid species) and lyso-PG (LPG, 9 lipid species), phosphatidic acid (PA, 6 lipid species) and lyso-PA (LPA, 1 lipid species) and phosphatidylinositol (PI, 5 lipid species) and lyso-PI (1 LPI species), as summarized in the Table 4. LC–MS spectra of these phospholipid classes and MS/MS data are shown in the Supplementary Materials (Table S1, Figures S5–S9). The relative percentage of each lipid species within each specific class is shown in Figure 4. The majority of PLs species included C14:0, C16-, C18- and C20-fatty acyl chains. The DoU of lipid species varied between 0 and 11, in the case of PC, between 0 and 8 in PE, between 0 and 9 in PG, between 5 and 9 in PA (that included C16- and C20-fatty acyl chains), between 0 and 10 in PI (that included C16- and C18-fatty acyl chains), between 0 and 6 in LPC and between 0 and 5 in LPE and LPG. Regarding the lyso-lipids LPI and LPA, the only lipid species identified were LPI (16:0), at *m*/*z* 571.2897 and LPA (20:4), at *m*/*z* 457.2362, respectively. The most abundant lipid species within each class included 36-, 38- and 40-carbons, in the case of PC, 32- and 34-carbons in PE, 36-carbons in PG, 40-carbons in PA and 34- and 42-carbons in PI. In the case of lyso-PLs, the most abundant lipid species included 16-,18-,20-carbons in LPC, 16- and 18-carbons in LPE and 16- and 20-carbons in LPG.

Classes PC and LPC were identified as [M + H]^+^ ions in the LC−MS spectra and fatty acyl composition was assigned by the identification of the carboxylate ions (RCOO−) in the LC−MS/MS of the [M + CH_3_OO]^–^ ions. The most abundant lipid species were PC (40:9), assigned as PC (20:4/20:5) at *m*/*z* 828.5542, and LPC (20:4) at *m*/*z* 544.3406, as observed in the Figure 4a,b and Table 4. Classes PE and LPE were identified as [M − H]^–^ and [M + H]^+^ ions in the LC−MS and the fatty acyl composition was assigned by the identification of the carboxylate ions (RCOO^−^) in the LC−MS/MS spectra. The most abundant lipid species were PE (32:1), identified as PE (16:1/16:0) at *m*/*z* 688.4932, and LPE (16:1) at *m*/*z* 452.2787, as observed in the Figure 4c,d and Table 4. The molecular species of PG and LPG were identified as [M − H]^–^ ions (Figure 4e–f). The most abundant were PG (36:5) at *m*/*z* 767.4878, identified as PG (20:5/16:0) and PG (20:4/16:1), and LPG (20:5) at *m*/*z* 529.2577. The hydroxy-lipids PG (36:5)OH and LPG(16-OH) were identified in these classes. Classes PA and PI were assigned in LC−MS and MS/MS spectra as [M − H]^−^ ions. In these classes, the most abundant lipid species were PA (40:8) at *m*/*z* 743.4660, identified as PA (20:4/20:4) (Table 4, Figure 4g), and PI (34:3) at *m*/*z* 831.5030, Table 4, Figure 4h). Concerning phosphoinositol ceramides (PI-Cer), the species were identified as [M − H]^−^ ions in the LC−MS spectra (Table 4, Figure 4i). LC–MS spectra of this class and MS/MS data are shown in Figure S10 in the Supplementary Materials. The eight lipid species identified included saturated- and unsaturated-sphingosine-bases and saturated and unsaturated hydroxylated acyl chains. The most abundant lipid species included 42-carbons PI-Cer. The most abundant PI-Cer (t42:2h) at *m*/*z* 920.6240 contained the long-chain base 18:1-phytosphingosine (trihydroxy sphingoid base) and the long monounsaturated hydroxy-fatty acid having 24-carbons (PI-Cer (t18:1/h24:1).

#### 2.2.4. Fatty Acids Profile of *Grateloupia turuturu*

The lipid extracts were further analysed for esterified fatty acids (FA) profiling and a total of 19 FA were identified by GC–MS after the transmethylation (Table 5, Figure S11). The FA acid profile calculated as relative abundance showed ca. 37.50% of saturated FA (SFA), ca. 16.70% of monounsaturated FA (MUFA) and ca. 49.51% of polyunsaturated FA (PUFA) (Table 5). The three fatty acids that contributed about 60% of the total FA content were palmitic acid (C16:0), the most abundant fatty acid which accounted for 60.48 ± 10.44 µg mg^−1^ extract, ca. 22.03% of the FA content (or 628.89 ± 47.70 µg g^−1^ DW biomass), eicosapentaenoic acid (EPA; C20:5*n*-3) which accounted for 57.18 ± 8.74 µg mg^−1^ extract, ca. 20.86% of FA content (or 595.19 ± 32.17 µg g^−1^ DW biomass), and arachidonic acid (AA, C20:4*n*-6) which accounted for 35.41 ± 5.76 µg mg^−^^1^ extract, ca. 12.91% of FA content (368.19 ± 20.56 µg g^−1^ DW biomass). Stearic acid (C18:0) and oleic acid (C18:1*n*-9) accounted for 5.44 % and 7.44 %, respectively, while other FAs, such as linoleic acid (C18:2*n*-6), linolenic acid (C18:3*n*-3) and octadecatetraenoic acid (C18:4), were less than 5% of total FA content. Other minor fatty acids were identified, including FA with odd-number carbon chains (e.g., C15:0 and C17:0). The total content of esterified fatty acids was 273.52 ± 34.49 µg mg^−1^ extract (2853.16 ± 139.33 µg g^−1^ DW biomass). 

Based on their FA profile, lipid extracts were evaluated in light of their nutritional value and potential health benefits not only by determining the fatty acids profile, but also by calculating health lipid indices, such as Σ PUFA/Σ SFA and Σ PUFA *n*-6/*n*-3 ratios, as well as nutritive value (NVI), atherogenic (AI), thrombogenic (TI), hypocholesterolemic/hypercholesterolemic (h/H) and the peroxidizability index (PI) ratios (Table 5). The ratios of Σ PUFA/Σ SFA and Σ PUFA *n*-6/*n*-3 ratios represented, respectively, 1.32 ± 0.04 and 0.87 ± 0.01. *Grateloupia turuturu* FA showed an *n*-6/*n*-3 ratio < 1 and an NVI of about 0.77 ± 0.03, mainly due to the highest proportion of C16:0. Both AI and TI were < 1, while the h/H index was 2.4 ± 0.12. In addition, the quality of the lipid extract expressed as the percentage of PI was 218.42 ± 30.47%.

### 2.3. Antioxidant Activity of the Lipid Extracts of Grateloupia turuturu 

**Figure 5:**
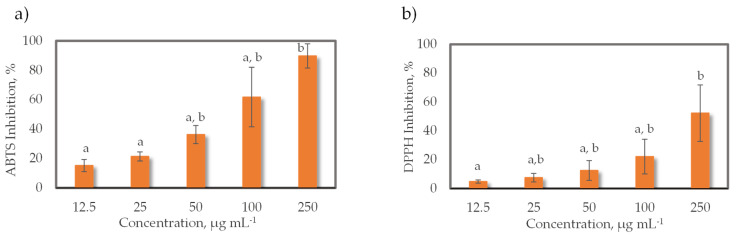
Free radical-scavenging activity (%) against the 2,2′-azino-bis-3-ethylbenzothiazoline-6-sulfonic acid radical cation (ABTS) (**a**) and the 2,2-diphenyl-1-picrylhydrazyl radical (DPPH) (**b**) from lipid extracts of *Grateloupia turuturu*. The concentration of the lipid extract was between 12.5 and 250 µg mL^−1^. Each value is expressed as the mean ± standard deviation (*n* = 4). Values with no same letter in each column have significant difference (*p* < 0.05).

A dose-dependent increase in the scavenging capacity was observed for all concentrations tested (Figure 5). For ABTS, a 50% inhibition (IC_50_) of the ABTS^●+^ radical was achieved at a concentration of 130.4 ± 52.4 μg mL^−1^, which represents a TE of 7.3 ± 3.7 µmol g^−1^. In the DPPH assay, the IC_50_ value could not be calculated because the percentage of antioxidant activity was less than 50%, so the IC_50_ values of the extracts were outside the tested concentration range. Thus, a 25% inhibition (IC_25_) of DPPH^●^ was obtained at a concentration of 129.1 ± 58.7 μg mL^−1^, representing a TE of 83.2 ± 39.6 µmol g^−1^. The best radical scavenging capacity was achieved at a concentration of 250 μg mL^−1^ in both ABTS (89.79% ± 8.22%) and DPPH (52.21% ± 19.57%). 

### 2.4. Cyclooxygenase 2 (COX 2) Enzyme Inhibitory Capacity of the Lipids Extracts of Grateloupia turuturu 

**Figure 6:**
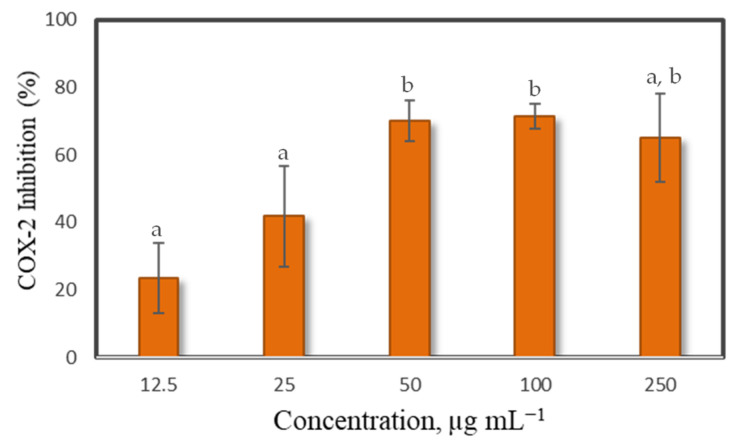
Cyclooxygenase 2 (COX-2) enzyme inhibitory capacity of lipid extracts of *Grateloupia turuturu* at a concentration between 12.5 and 250 µg mL^−1^. Each value is expressed as a percentage mean ± standard deviation (*n* = 4). Values with no same letter in each column have significant difference (*p* < 0.05).

All the concentrations of lipid extracts tested showed the capacity to inhibit COX-2 activity in vitro, although showing only a dose-dependent response between 12.5 and 50 µg mL^−1^ of lipid extract (Figure 6). However, a concentration of ca. 33 µg mL^−1^ was able to inhibit the PG2 production by 50%, demonstrating that the extract has a potential anti-inflammatory activity.

## 3. Discussion

*Grateloupia turuturu* is widely consumed in Asia [39] and its nutritional value is evidenced by the high proportion of proteins and *n*-3 fatty acid EPA (C20:5, *n*-3) that it displays [13,40,41]. In Europe, *G. turuturu* is yet to be explored, despite having EPA as one of its main fatty acids in the lipid pool, much as *Palmaria palmata*, a red seaweed commercially used and appreciated in western countries [42].

*Grateloupia turuturu*, studied in the present work, contained 26.26 ± 0.69 g 100g^−1^ DW of protein, in line with the levels reported for other red seaweeds [13,14]. This value is close to the maximum concentration indicated by Denis et al. for the specimens sampled on the Brittany coast [13]. The total lipid content of *G. turuturu* was determined and accounted for 0.88 ± 0.25 g 100 g^−1^ of biomass in dry weight (DW). This result is consistent with data from the literature (ca. 0.7%, up to 4.0% DW) [14,17,43,44] and in line for that of other red seaweeds [28,45,46,47]. Low fat and high protein content make red seaweeds a promising alternative for non-animal proteins [48,49]. In addition, the obtained C/N ratio of 8.30 ± 0.19 (Table 1) represents a value quite similar to those of nitrophilic red seaweeds, values below the proposed critical limit of 10 [50]. This value expresses a balanced nutrient status of *G. turuturu* [51,52] and corresponds to the trend high protein–low lipid relationship as found in our study.

The nutritional value of *G. turuturu* was also demonstrated by the profiling of FAs and the calculation of nutritional indices (Table 5). The saturated FA level was lower than that previously described for *G. turuturu* from Brittany [13] and within the range reported by Hotimchenko for *G. turuturu* sampled in the Sea of Japan [44]. Palmitic acid (C16:0) was the most abundant FA (ca. 22.03%) followed by C20:5*n*-3 (ca. 20.86%) (Table 5), as previously described by Rodrigues et al. [15] and Hotimchenko [44], but different from those reported by Denis et al. [13] and Kendel et al. [16,19], who obtained a 2.0-fold relationship of C16:0 *versus* C20:5*n*-3 FA in *G. turuturu*. This difference was attributed to both environmental conditions and genetic status of the seaweed [13]. 

Eicosapentaenoic acid is the main PUFA in the lipid extract of *G. turuturu* (5.7 g per 100 g^−1^ of lipid extract (ca. 0.06 g of EPA per 100 grams of DW biomass, or ca. 0.01 g of EPA per 100 grams of fresh biomass)), that can be proximate to certain *n*-3 in fish such as dogfish [53]. According to U.S. Food and Drug Administration (FDA, https://www.fda.gov/food, accessed on 13 May 2021), new recommendations for the consumption of EPA and DHA claim that a daily dose of, at least, 0.8 g of EPA and DHA (combined total) provides a beneficial effect on health. Taking into account the EPA content of the lipid extract of *G. turuturu*, ca. 14 g of lipid extract (1.4 times a tablespoon) would provide proximately 0.8 g of EPA per serving. The nutritional value of *G. turuturu* extracts was corroborated by the PUFA/SFA ratio calculated in the present work, as it was greater than the recommended threshold 0.45, as well as by its *n*-6/*n*-3 ratio (<1). These values are consistent with those previously reported in *Grateloupia* spp. [15] and other red seaweeds [46] and have been associated with human health benefits, such as the prevention of noncommunicable and cardiovascular diseases [54,55]. The nutritive index (NVI) obtained (0.77 ± 0.03) was clearly driven by the higher proportion of C16:0, compared to C18:0 and C18:1 FA. The calculated AI and TI were < 1 and < 0.5, respectively, as recommended to be protective against coronary artery disease [56]. The values of AI and TI were within the reported for the red seaweeds *Porphyra dioica* [29] and *P. palmata* [46] and marine fish [57]. The ratio between hypocholesterolemic and hypercholesterolemic fatty acids (h/H index) is also within the values reported for marine fish [57]. Finally, the quality of lipid extract and the stability of PUFA included in food and its vulnerability to oxidation, evaluated by calculating the peroxidizability index (PI percentage), achieved a value of 218.42 ± 30.47%, thus expressing a balanced relation between the required amount and type of PUFA that guarantees a protective effect for coronary disease. Therefore, *G. turuturu* can be used in the food industry applications contributing to dietary recommendations of SFA and PUFA intake (http://www.fao.org, accessed on 13 May 2021). 

Polar lipids from seaweeds are considered high-value lipids with beneficial health effects fostering seaweed valorization. The lipidome of *G. turuturu* was identified here for the first time using an approach based on liquid chromatography–mass spectrometry, which allowed the identification of 205 lipid species distributed over glycolipids, betaine lipids, phospholipids and phosphoinositol ceramides PI-Cer (Table 2, Table 3 and Table 4). The complex lipids (polar lipids) from seaweeds are gaining a new interest because they are the key carriers of PUFAs and feature bioactive properties [58,59]. The classes of glycolipid were previously identified for *G. turuturu* [19] and *G. jilicina* [60] by using preparative thin-layer chromatography and the PL was determined by HPLC coupled to an evaporative light scattering detector [16]. Betaine lipids and PI-Cer classes were identified in the lipidome of *G. turuturu* for the first time in the present study but have already been described in other red seaweeds [26], such as *Porphyra dioica* [29] and *Palmaria palmata* [46]. It is also worth referring that PI-Cer lipids are considered to be biomarkers of Rhodophyta [46,61,62]. 

Several glycolipids and phospholipids identified in the *G. turuturu* lipidome were esterified to EPA, including the most abundant lipid species in most classes, as described for *P. palmata* [46]. Marine polar lipids are considered to be important carriers of *n*-3 fatty acids with higher *n*-3 PUFA levels than triglycerides (TAG) [63,64]. The bioavailability of PUFAs comprising *n*-3 FAs is also considered to be higher when these FAs are esterified into polar lipids, namely PL, compared to TAG [65]. In addition, polar lipids such as glycolipids and phospholipids from seaweeds are recognized by their wide range of bio functionalities [66] and the interest in these healthy and bioactive molecules is growing. Polar lipids can be used as an ingredient for food fortification in PUFA and in functional food, or used as emulsifying agents in the food industry [63,64,65,66].

In this study, the antioxidant and cyclooxygenase-2 inhibitory actions of the lipid extract of *G. turuturu* were tested. The concentrations necessary to inhibit the activity of the DPPH radical by 20% are of the same order as that reported for *G. gracilis* (IC_20_ 119.5 ± 1.8 µg mL^−1^) and *P. palmata* (IC_20_ 119.6 ± 8.0 µg mL^−1^) [67]. Our results reveal that a polar lipid extract of *G. turuturu* features antioxidant activity at low concentrations. The scavenging activity of *G. filicina* extracts, obtained using other organic solvent systems (e.g., ethyl acetate, chloroform, or acetone) [35], was a 50% inhibition achieved at higher concentrations (2 mg mL^−1^ of extract). In the same study, chloroform extracts had higher antioxidant activity than commercial antioxidants, such as BHA (butylated hydroxyanisole), BHT (butylated hydroxytoluene) and a-tocopherol [35]. Overall, *G. turuturu* lipid extracts exhibit antioxidant activity and can be used as natural antioxidants for food and feed, as a dietary supplement to nutraceuticals, or as an active ingredient in functional foods or cosmeceuticals promoting health and prevention against damages caused by free radicals. Other potential applications include food processing industries to prevent oxidation and replace synthetic antioxidants. The antioxidant effects of natural products are necessary both for health and wellbeing to counter oxidative stress [68,69], but also for the preservation and packaging of food to increase shelf life through reduction of lipid peroxidation [70].

The ability of lipid extracts from *G. turuturu* to inhibit COX-2 and, thus, reducing the formation of PGH2, was herein demonstrated reaching 50% inhibitory effect at 33 µg mL^−1^ (Figure 6). Our findings show that these extracts were more effective than those from other red seaweeds, such as *P. palmata and P. dioica*, which achieved more than 80% inhibition of COX-2 using 500 µg mL^−1^ of lipid extract [67]. Cyclooxygenase-2 (COX-2) is a key enzyme in fatty acid metabolism that is upregulated in inflammation. COX-2 is induced by proinflammatory cytokines and enhances the synthesis of prostaglandins, which stimulates inflammation response [54]. Targeted inhibition of COX-2 is a promising approach to inhibit inflammation, with phytonutrients and phytochemicals holding the potential to act in this regulation [71]. These results are in line with those reported by Yang et al. [37], who showed the anti-inflammatory effect of ethyl acetate extracts from *G. elliptica* on the inhibition of prostaglandin E2 (PGE2) in a macrophages cell line (IC_50_ values of the same order found in this study). In the same work, inhibition of the production of pro-inflammatory mediators such as nitric oxide (NO) was also reported. Even though no lipid species have so far been attributed to this specific activity, several lipid species that were well-represented in the lipid extracts of *G. turuturu* analyzed in the present work (MGDG and DGDG contained (20:5/20:5), (20:5/20:4), (18:4/16:0), (20:4/16:0), (20:5/16:0) and (20:5/14:0), SQDG (20:5/14:0), PG (20:5/16:0), PG (20:5/16:1) and PC (20:5/20:5)), have been reported to be related with anti-inflammatory activity on the downregulation of iNOS in macrophages [72,73]. These lipid species were isolated from extracts of red seaweeds *Chondrus crispus* and *P. palmata.* All of those lipid species contained C20:5(*n*-3) FA and showed higher activity than the free FA C20:5(*n*-3), suggesting that a higher bioactivity was likely due to the polar head of these molecules. These characteristics support per se the growing interest in polar lipids from seaweeds, principally those originating from red seaweeds [67]. Particularly, GLs bearing *n*-3 PUFA have been related to bioactive properties, such as antibacterial, antitumoral and antiviral activities, enhancing the pharmacological potential of these compounds [58]. Recent research associated with galactolipids “takes us back to our origins” by reminding that eating vegetables provides access to *n*-3 FA and contributes to the balance anti-inflammatory (*n*-3) and pro-inflammatory (*n*-6) fatty acids [74]. It is known that PLs display anti-inflammatory, anti-oxidant, anti-fibrogenic, anti-apoptotic, membrane-protective and lipid-regulating effects, with a positive impact on several diseases, apparently without significant side effects [63]. Although the market for marine PLs is still in its early stages, there is a growing trend to use marine PLs to supply *n*-3 PUFA for the global food and dietary supplements market [63]. All beneficial effects of marine lipids should be considered venues for future research.

In summary, *G. turuturu* lipids can be used in the food industry, using the seaweed biomass as an ingredient for food fortification and functional foods (e.g., dairy products or supplements), or by using the lipid extracts as ingredients for nutraceutical, pharmaceutical and cosmeceutical applications, providing valuable products to be explored for commercialization [75]. Lipidomics can therefore be used for screening the biomass with the best composition in polar lipids richer in PUFA and with the most promising bioactivities, allowing the selection of the seaweed strains for commercial exploitation [6,29].

Overall, the findings reported in the present work add value to *G. turuturu* and contribute to the development of blue bioeconomy.

## 4. Materials and Methods

### 4.1. Reagents

All chemicals and reagents were purchased from Fisher Scientific Ltd. (Loughborough, UK), unless otherwise specified [71]. Lipid standards were purchased from Avanti Polar Lipids, Inc. (Alabaster, AL, USA) [71]. Ammonium acetate and 6-hydroxyl-2,5,7,8-tetramethylchromane-2-carboxylic acid (Trolox) were purchased from Sigma-Aldrich (St Louis, MO, USA). 2,2-Diphenyl-1-picrylhydrazyl (DPPH) was purchased from Aldrich (Milwaukee, WI, USA). 2,2′-Azino-bis-3-ethylbenzothiazoline-6-sulfonic acid diammonium salt (ABTS) was obtained from Fluka (Buchs, Switzerland). Milli-Q water (Synergy, Millipore Corporation, Billerica, MA, USA) was used.

### 4.2. Collection of Macroalgae and Preparation of Biomass 

The specimens of the red seaweed *G. turuturu* Yamada were hand-harvested in Ria de Aveiro coastal lagoon (Gafanha da Nazaré, Portugal, 40°39′ N, 8°43′ W) in February 2017 (winter). Samples were rinsed with fresh water, cleaned of epiphytes, if present, and oven-dried at 30 °C in an air tunnel (up to ca. 13% moisture content was achieved). Biomass of at least 5 specimens was used, five replicates of ca. 200 mg to extract total lipids and three replicates of 2 mg to determine the C/N ratio.

### 4.3. Proximate Elemental Composition Analysis

Elemental analysis (C and N) (2 mg × 3 replicates) was performed using an elemental analyzer (Leco TruspecMicro CHNS 630-200-200) at the combustion furnace temperature of 1075 °C and an afterburner temperature of 850 °C. The carbon-to-nitrogen ratio (C/N) was calculated through tissue C (g 100 g^−1^ dry weight biomass, DW)/tissue N content (g 100 g^−1^ DW) ratio (excluding moisture from sample weight). The nitrogen–protein conversion factor 6.25 was used to estimate protein content.

### 4.4. Lipid Extraction

The lipid content was determined by gravimetry. Lipids were extracted from weighed samples (200 mg each, for a total of 5 replicates) by adding 3 mL of methanol: chloroform mixture (2:1, *v*/*v*) to each sample replicate in glass tubes with Teflon-lined screw caps (modified Bligh and Dyer method used in the Marine Lipidomics Laboratory [28,71]). The organic phase was collected first and the remaining biomass residue was re-extracted twice. Water (2 mL) and chloroform (2 mL) were added to the collected total organic phase and the lower organic phase was collected for drying under a stream of nitrogen gas. The content of total lipid extract was estimated by gravimetry and stored at –20 °C, under a nitrogen atmosphere, before analysis. The five replicates were analyzed by LC–MS.

### 4.5. Hydrophilic Interaction Liquid Chromatography−High Resolution Mass Spectrometry (HILIC−MS) and Tandem Mass Spectrometry (MS/MS)

Analysis of lipid extracts was performed in a high-performance liquid chromatography (HPLC) Ultimate 3000 Dionex (Thermo Fisher Scientific, Bremen, Germany) system with an autosampler and coupled online to the Q-Exactive^®^ hybrid quadrupole Orbitrap^®^ mass spectrometer (Thermo Fisher Scientific, Waltham, MA, USA). An Ascentis Si column (150 mm × 1 mm, 3 μm, Sigma-Aldrich, St. Louis, MO, USA) and a two-phases-solvent system (mobile phase A, acetonitrile/methanol/water (50:25:25, *v*/*v*/*v*) with 1 mM ammonium acetate; mobile phase B, acetonitrile/ methanol (60:40, *v*/*v*) with 1 mM ammonium acetate) were employed for the separation of lipids by HILIC-chromatography, as previously described [71]. The injection volume was 5 μL of each sample containing 10 μg of lipid extract, a volume of 4 μL of a mixture of phospholipid standards mix, described in da Costa et al. (2020) [71], and 86 μL of mobile phase B.

The MS employed was equipped with Orbitrap technology and was operated using a positive/negative switching toggle between positive (electrospray voltage of 3.0 kV) and negative (electrospray voltage of 2.7 kV) ion modes, with a capillary temperature of 250 °C and sheath gas flow of 15 arbitrary units (a.u.). Mass spectra were acquired using data-depending acquisition (DDA) mode, with cycles of one full-scan mass spectrum (mass resolving power of 70,000 full width at half-maximum, automatic gain control target of 1 × 10^6^, 200–1600 *m*/*z* scan range) and ten data-dependent MS/MS scans (resolution of 17,500 width at half-maximum and automatic gain control target of 1 × 10^5^) with the dynamic exclusion of 60 s and intensity threshold of 1×10^4^, repeated continuously throughout the experiments. Tandem MS fragmentation was performed by higher-energy collisional dissociation (HCD), using stepped normalized collision energy ranging between 25, 30 and 35 eV. Data acquisition was carried out using the Xcalibur data system (V3.3, Thermo Fisher Scientific, MA, USA). 

### 4.6. Data Analysis and Lipids Identification

Data acquisition was performed using the Xcalibur data system (V3.3, Thermo Fisher Scientific, Waltham, MA, USA). Peak integration and HPLC–MS data assignments were performed using MZmine 2.42 and the predefined parameters for data processing, as described in similar approaches published by the Marine Lipidomics Group [30]. Lipid identification was performed by matching the LC retention time (shown in the total ion chromatograms, Supplementary Materials, Figure S1), with the assignment of the molecular ions observed in the LC–MS spectra (Figures S2–S8), with an accuracy of mass measurements < 5 ppm (Table 2, Table 3 and Table 4). Additionally, manual analysis of MS/MS spectra confirmed the identity of the polar head and fatty acyl composition of most of the identified lipid the molecular species (Supplementary Materials, Table S1 and Figures S2–S10). Typical fragmentation rules used to generate the assignments were included [71]. The normalization of lipid species was completed by dividing the exported values of integrated peak areas of each lipid species by the value of the peak area of a standard lipid species with the closest retention time.

### 4.7. Analysis of Fatty acid by Gas Chromatography–Mass Spectrometry (GC–MS)

Fatty acid methyl esters (FAMEs) were prepared by an alkaline well-established methodology [28,71,76]. A 70 µg aliquot of the lipids extracted from each sample was derivatized in a screw cap glass tube. Transesterification was performed by transferring 1 mL of a standard of methyl nonadecanoate (C19:0, 74208, Sigma-Aldrich, St. Louis, MO, USA) solution in hexane (1.27 μg mL^−1^) and 200 µL of KOH 2 M methanol. An aliquot of 600 μL of the upper phase was collected, dried under a stream of nitrogen gas and, subsequently, 120 μL of hexane was added to prepare the final solution for GC–MS analysis.

A 2 μL subsample of the FAME solution in hexane obtained from the derivatization was injected in an Agilent 8860 GC System gas chromatograph with GC 5977B Network Mass Selective Detector operating at 70 eV at 250 °C and equipped with a DBFFAP (Agilent 123-3232, 30 m × 320 µm × 0.25 µm) column. GC–MS was equipped with an auto sampler with a splitless injector at 220 °C. The separation of FAME was carried out with helium being used as the carrier gas (constant flow rate of 1.4 mL min^−1^) and using a temperature program for the column starting at 80 °C during 2 min and increasing to 160 °C at 25 °C/min, heating up to 210 °C at 2 °C/min, then to 225 °C at 20 °C/min and holding for 20 min. The system employed includes a Mass Selective Detector operating in Electron Ionization (EI) mode at 70 eV and scanning the mass range *m*/*z* 50–550 in a 1 s cycle in a full scan mode acquisition. Analyses were always replicated (at least *n* = 3). Methyl esters were identified using the software Agilent MassHunter Qualitative10.0, supported by NIST2014 mass spectral library, by comparing their retention time and MS spectra with those of Sigma-Aldrich standards (37 Component FAME Mix, Sigma-Aldrich) and by MS spectra comparison with online databases (AOCS lipid library). Quantitative analysis of FA was achieved from calibration curves of each methyl ester of FA from a FAME mixture (Supelco 37 Component FAME Mix, CRM47885, Sigma Aldrich, St. Louis, MO, USA), analyzed by GC–MS under the same conditions of extracts, with results being expressed as µg mg^−1^ of extract and µg g^−1^ of dry biomass. The relative amounts of FAs were calculated using the ratio of the amount of each FAME and the sum of all FAMEs identified; results are expressed as means (%, *w*/*w*). Nutritional, health and quality indices nutritive value (NVI), atherogenic (AI), thrombogenic (TI), hypocholesterolemic/hypercholesterolemic (h/H) and peroxidizability indices (PI) were determined according to the literature [56].

### 4.8. 2,2′-Azino-bis-3-Ethylbenzothiazoline-6-Sulfonic Acid Radical Cation Assay—ABTS Radical Scavenging Activity

The antioxidant scavenging activity against the 2,2′-azino-bis-3-ethyl benzothiazoline-6-sulfonic acid radical cation (ABTS^●+^) was evaluated using a previously described method [77]. The ABTS radical solution (3.5 mmol L^−1^) was prepared. This mixture was kept for 16 h in the dark at room temperature; then, it was diluted in ethanol to obtain an absorbance value of ~0.9, measured at 734 nm using a UV-vis spectrophotometer (Multiskan GO 1.00.38, Thermo Scientific, Hudson, NH, USA). Radical stability was determined as reported by Santos et al. [77]. For an evaluation of the radical scavenging potential, a volume of 150 µL of each lipid extract of *G. turuturu* (12.5-250 µmol L^−1^ in ethanol, *n* = 4), or 150 µL of Trolox standard solution (10–75 µmol L^−1^), was placed in each well, followed by the addition of 150 µL of ABTS^●+^ diluted solution. Control lipid assays were prepared by replacing 150 µL of ABTS^●+^ diluted solution with 150 µL of ethanol. The % of the ABTS radical remaining was determined according to Equation (1); free radical-scavenging activity of samples was calculated as the percentage of inhibition of the ABTS radical (Equation (2)). The concentration of samples reducing 50% of the ABTS radical after 120 min (IC_50_) were calculated by linear regression using the concentration of samples and the percentage of the inhibition curve. The activity was expressed as Trolox Equivalents (TE, µmol Trolox/g of sample) according to Equation (3). The significant differences (*p* < 0.05) calculated by Kruskal–Wallis with Dunn’s multiple comparisons were used to analyze the results between groups (GraphPad Prism 8).

% ABTS remaining = (Abs samples after incubation time/Abs sample at the beginning of reaction) × 100(1)

% Inhibition = ((Abs ABTS − (Abs samples − Abs control))/Abs ABTS) × 100(2)

(TE = IC_50_ Trolox (µmol L^−1^) × 1000/IC_50_ of samples (µg mL^−1^) (3)

### 4.9. 2,2-Diphenyl-1-Picrylhydrazyl Radical Assay—DPPH Radical Scavenging Activity 

The antioxidant scavenging activity against the 2,2-diphenyl-β-picrylhydrazyl radical (DPPH^●^) was evaluated using a previously described method [77]. A stock solution of DPPH^●^ in ethanol (250 µmol L^−1^) was prepared and diluted to provide a working solution with an absorbance value of ~0.9, measured at 517 nm using a UV-Vis spectrophotometer (Multiskan GO 1.00.38, Thermo Scientific, Hudson, NH, USA). The evaluation of the radical stability was determined as previously reported [77]. For evaluation of the radical scavenging potential, a volume of 150 µL of each lipid extract of *G. turuturu* (12.5–250 µmol L^−1^, *n* = 4), or 150 µL of Trolox standard solution (10–75 µmol L^−1^), was placed in each well, followed by the addition of 150 µL of a DPPH^●^ diluted solution and, again, an incubation period of 120 min before measuring absorbance at 517 nm every 5 min. The % of the DPPH radical remaining was calculated according to Equation (4); free radical-scavenging activity of samples was determined as the percentage of inhibition of the DPPH radical (Equation (5)); the concentration of samples reducing 25% of the DPPH radical after 120 min (IC_25_) was calculated by linear regression using the concentration of samples and the percentage of the inhibition curve. The activity expressed, as TE (µmol Trolox g^−1^ of sample), was determined (Equation (6)). The significant differences (*p* < 0.05) calculated by Kruskal–Wallis with Dunn’s multiple comparisons were used to analyze the results between groups (GraphPad Prism 8).

% DPPH remaining = (Abs samples after incubation time/Abs sample at the beginning of reaction) × 100(4)

% Inhibition = ((Abs DPPH − (Abs samples − Abs control))/Abs DPPH) × 100(5)

TE = IC_25_ Trolox (µmol L^−1^) × 1000/IC_25_ of samples (µg mL^−1^) (6)

### 4.10. Cyclooxygenase 2 (COX 2) Enzyme Inhibitory Capacity of Lipid Extract

The inhibition potential against COX-2 was carried out by enzyme immunoassay (EIA) kit (catalogue No. 701080, Cayman Chemical Company, Ann Arbor, MI, USA), as described by the manufacturer (https://www.caymanchem.com/pdfs/701080.pdf, accessed on 6 March 2021) [67,71]. Lipid extracts were dissolved in 100% DMSO and five concentrations ranging between 12.5 and 250 µg mL^−1^ were tested. The amount of prostaglandin F2α generated from AA in the cyclooxygenase reaction was determined by spectrophotometry at 412 nm using a Multiskan GO 1.00.38 (Thermo Scientific, Hudson, NH, USA). The results are expressed as a percentage of inhibited COX-2. The significant differences (*p* < 0.05) calculated by Kruskal–Wallis with Dunn’s multiple comparisons were used to analyze the results between groups (GraphPad Prism 8).

## 5. Conclusions

The present work provides the first insight on the *G. turuturu* lipidome, its nutritional value and its anti-oxidant and anti-inflammatory potential. The lipid and protein composition of this red seaweed, the profiles of fatty acids and lipid species and the health lipid indices determined demonstrated the nutritional value of *G. turuturu*. The biomass of this red seaweed can therefore be considered as a potential source for food, feed and other high-end uses. For industrial applications, structural characterization efforts are essential for the valorization of these lipids. The antioxidant free radical scavenging potential and cyclooxygenase-2 inhibitory activity were investigated and activities were achieved at low concentrations of extracts. The lipid extracts of *G. turuturu* constitute an interesting offer of natural and effective molecules to fight against pathological complications linked to free radicals and to be used in industry. Overall, our findings will likely inspire future studies targeting *G. turuturu* as a source of nutritive biomass and bioactive molecules for nutraceutical, pharmaceutical and cosmeceutical applications.

## Figures and Tables

**Table 1:** Content of lipid, tissue carbon, tissue nitrogen and proteins expressed in g 100 g^−1^ of dry weight biomass (DW) and carbon-to-nitrogen ratio (C/N) of the *Grateloupia turuturu* biomass (means ± SD: ^a^ *n* = 5, ^b^ *n* = 3).

Compound	Content
Lipid content (g 100 g^−1^ DW) ^a^	0.88 ± 0.25
C (g 100 g^−1^ DW) ^b^	34.88 ± 0.31
N (g 100 g^−1^ DW) ^b^	4.20 ± 0.11
C/N	8.30 ± 0.19
Protein content (N × 6.25) (g 100 g^−1^ DW)	26.26 ± 0.69

**Table 2:** *Grateloupia turuturu* glycolipids species identified by HILIC–LC–MS and MS/MS: type of adduct, lipid identity, calculated and observed mass, error, fatty acyl composition confirmed by MS/MS and formula. MGDG, MGMG, DGDG, DGMG were identified as [M + NH_4_]^+^ ions, while SQDG and SQMG were identified as [M – H]^−^ ions. The most abundant species are indicated in italics and in bold. C represents total carbon atoms and N represents the double bonds of fatty acid substituents. Fatty acyl chains were identified by MS/MS (a, assignments by mass accuracy, but without MS/MS; b, assignments by mass accuracy, MS/MS with diagnostic ions confirming the lipid class, but without information on fatty acyl chains). The representation C:N/C:N signifies the attribution of fatty acyl to the position sn-1/sn-2 according to the nomenclature of LIPIDMAPS (www.lipidmaps.org, accessed on 25 April 2021).

Lipid Species (C:N)	Calculated *m*/*z*	Observed *m*/*z*	Error (ppm)	Fatty Acyl Chains (C:N)	Formula
**MGDG identified as [M + NH_4_]^+^**					
MGDG(30:4)	712.5000	712.5028	3.9169	a	C39H70NO10
MGDG(34:7)	762.5156	762.5158	0.1854	18:3_16:4	C43H72NO10
MGDG(34:2)	772.5933	772.5896	−4.8099	a	C43H82NO10
MGDG(34:1)	774.609	774.6103	1.6896	18:1_16:0	C43H84NO10
MGDG(36:9)	786.5156	786.5177	2.6952	a	C45H72NO10
MGDG(36:8)	788.5313	788.5308	−0.5953	20:4_16:4	C45H74NO10
MGDG(36:6)	792.5625	792.5619	−0.7124	a	C45H78NO10
MGDG(36:5)	794.5782	794.5780	−0.1981	20:5_16:0	C45H80NO10
MGDG(36:4)	796.5933	796.5926	−0.8679	20:4_16:0	C45H82NO10
MGDG(36:3)	798.6095	798.6074	−2.6311	20:3_16:0	C45H84NO10
MGDG(38:8)	816.5626	816.5621	−0.5736	a	C47H78NO10
MGDG(38:7)	818.5782	818.5800	2.135	a	C47H80NO10
MGDG(38:5)	822.6095	822.6100	0.5639	a	C47H84NO10
***MGDG(40:10)***	***840.5626***	***840.5633***	***0.8243***	***20:5/20:5***	***C49H78NO10***
MGDG(40:2)	856.6878	856.6879	0.2035	a	C49H94NO10
MGDG(44:10)	894.6095	894.6102	0.7878	a	C53H84NO10
**MGMG identified as [M + NH_4_]^+^**					
MGMG(14:0)	482.3329	482.333	0.2488	a	C23H48NO9
MGMG(16:4)	502.3016	502.3015	−0.2407	a	C25H44NO9
MGMG(16:3)	504.3173	504.319	3.3957	a	C25H46NO9
MGMG(16:2)	506.3329	506.3321	−1.6151	a	C25H48NO9
MGMG(16:1)	508.3486	508.349	0.9149	a	C25H50NO9
***MGMG(16:0)***	***510.3642***	***510.3637***	***−0.8972***	***16:0***	***C25H52NO9***
MGMG(18:4)	530.3329	530.3342	2.3898	a	C27H48NO9
MGMG(18:3)	532.3486	532.3497	2.1221	a	C27H50NO9
MGMG 18:2)	534.3642	534.3639	−0.5996	a	C27H52NO9
MGMG(18:1)	536.3799	536.3793	−1.1276	a	C27H54NO9
***MGMG(20:5)***	***556.3486***	***556.3488***	***0.3521***	***20:5***	***C29H50NO9***
MGMG(20:4)	558.3642	558.3646	0.7432	a	C29H52NO9
MGMG(20:3)	560.3799	560.3807	1.5291	a	C29H54NO9
**DGDG identified as [M + NH_4_]^+^**					
DGDG(32:2)	906.6154	906.6158	0.4273	16:2_16:0	C47H88O15N
DGDG(32:1)	908.6310	908.6315	0.5304	18:1_14:0; 16:1_16:0	C47H90O15N
DGDG(32:0)	910.6467	910.6465	−0.2435	a	C47H92O15N
DGDG(34:8)	922.5528	922.5488	−4.3802	a	C49H80O15N
DGDG(34:7)	924.5684	924.5639	−4.8671	a	C49H82O15N
DGDG(34:4)	930.6154	930.6146	−0.824	a	C49H88O15N
DGDG(34:3)	932.6310	932.6304	−0.6395	18:3_16:0	C49H90O15N
DGDG(34:2)	934.6467	934.6468	0.0566	18:2_16:0	C49H92O15N
DGDG(34:1)	936.6623	936.6627	0.422	18:1_16:0	C49H94O15N
***DGDG(36:5)***	***956.6310***	***956.6315***	***0.5293***	***20:5_16:0***	***C51H90O15N***
DGDG(36:2)	962.6780	962.6777	−0.2759	18:1/18:1	C51H96O15N
DGDG(38:5)	984.6623	984.6647	2.4117	a	C53H94O15N
DGDG(40:10)	1002.6154	1002.6164	0.9974	20:5/20:5	C55H88O15N
**DGMG identified as [M + NH_4_]^+^**					
DGMG(16:1)	670.4014	670.4020	0.8999	a	C31H60NO14
***DGMG(16:0)***	***672.4170***	***672.4174***	***0.5239***	***16:0***	***C31H62NO14***
DGMG(18:1)	698.4327	698.4332	0.7083	18:1	C33H64NO14
DGMG(20:5)	718.4014	718.4012	−0.3122	a	C35H60NO14
**SQDG identified as [M − H]^−^**					
SQDG(28:0)	737.4510	737.4518	1.0919	12:0_16:0	C37H69O12S
SQDG(30:2)	761.4510	761.4519	1.2214	a	C39H69O12S
SQDG(30:1)	763.4666	763.4668	0.2246	14:0_16:1	C39H71O12S
SQDG(30:0)	765.4823	765.4828	0.7155	14:0_16:0	C39H73O12S
SQDG(32:4)	785.4510	785.453	2.5189	14:0_18:4	C41H69O12S
SQDG(32:3)	787.4666	787.4658	−1.0089	a	C41H71O12S
SQDG(32:2)	789.4823	789.4833	1.2813	16:2_16:0	C41H73O12S
SQDG(32:1)	791.4979	791.4991	1.4932	16:1_16:0	C41H75O12S
SQDG(32:0)	793.5136	793.5147	1.3591	16:0/16:0	C41H77O12S
SQDG(34:5)	811.4666	811.4701	4.2331	14:0_20:5	C43H71O12S
SQDG(34:3)	815.4979	815.4986	0.8394	18:3_16:0	C43H75O12S
SQDG(34:2)	817.5136	817.5151	1.9049	a	C43H77O12S
SQDG(34:1)	819.5292	819.5308	1.9621	18:1_16:0	C43H79O12S
SQDG(34:0)	821.5449	821.5459	1.2441	a	C43H81O12S
SQDG(36:6)	837.4823	837.4844	2.5124	b	C45H73O12S
***SQDG(36:5)***	***839.4979***	***839.4989***	***1.1462***	***20:5_16:0***	***C45H75O12S***
SQDG(36:5)OH	855.4939	855.4943	0.4953	20:5−OH_16:0	C45H75O12SO
SQDG(38:7)	863.4979	863.4996	1.9862	20:4_18:3	C47H75O12S
SQDG(38:5)	867.5292	867.5298	0.7138	20:5_18:0	C47H79O12S
SQDG(40:10)	885.4823	885.4837	1.5835	20:5/20:5	C49H73O12S
**SQMG identified as [M − H]^−^**					
SQMG(14:0)	527.2526	527.2540	2.7046	a	C23H43O11S
SQMG(16:1)	553.2683	553.2701	3.267	a	C25H45O11S
***SQMG(16:0)***	***555.2839***	***555.2849***	***1.7351***	***16:0***	***C25H47O11S***
SQMG(18:1)	581.2996	581.3024	4.8607	a	C27H49O11S
SQMG(18:0)	583.3152	583.3166	2.3402	18:0	C27H51O11S
SQMG(20:5)	601.2683	601.2696	2.2023	a	C29H45O11S
SQMG(20:4)	603.2839	603.2851	2.0425	a	C29H47O11S
SQMG(24:0)	667.4091	667.4117	3.9262	a	C33H63O11S

**Table 3:** *Grateloupia turuturu* betaine lipids species identified by HILIC–LC–MS and MS/MS: type of adduct, lipid identity, calculated and observed mass, error, fatty acyl composition confirmed by MS/MS and formula. DGTS and MGTS were identified as [M + H]^+^ ions. The most abundant species are indicated in italics and in bold. C represents total carbon atoms and N represents the number of double bonds of fatty acid substituents. Fatty acyl chains were identified by MS/MS (a, assignments by mass accuracy and unequivocal molecular formula, but without MS/MS; b, assignments by mass accuracy, MS/MS diagnostic ions information to confirm the lipid class, but without information on fatty acyl chains). The representation C:N/C:N signifies the attribution of fatty acyls to the position sn-1/sn-2 according to the nomenclature of LIPIDMAPS (www.lipidmaps.org, accessed on 25 April 2021).

Lipid Species (C:N)	Calculated *m*/*z*	Observed *m*/*z*	Error (ppm)	Fatty Acyl Chains (C:N)	Formula
**DGTS identified as [M+H]^+^**					
DGTS(32:2)	708.5778	708.5792	1.9979	a	C42H78O7N
DGTS(32:1)	710.5935	710.5939	0.5111	a	C42H80O7N
DGTS(32:0)	712.6091	712.6083	−1.1556	a	C42H82O7N
DGTS(34:4)	732.5778	732.5784	0.7925	16:0_18:4	C44H78O7N
DGTS(34:3)	734.5935	734.5923	−1.6029	a	C44H80O7N
***DGTS(34:2)***	***736.6091***	***736.6101***	***1.3137***	***18:1_16:1***	***C44H82O7N***
DGTS(34:1)	738.6248	738.6238	−1.4067	a	C44H84O7N
DGTS(36:8)	752.5465	752.5482	2.2335	a	C46H74O7N
DGTS(36:7)	754.5622	754.5614	−1.0468	a	C46H76O7N
DGTS(36:5)	758.5935	758.5942	0.9613	a	C46H80O7N
DGTS(36:4)	760.6091	760.6072	−2.4467	a	C46H82O7N
DGTS(36:3)	762.6248	762.6238	−1.2584	a	C46H84O7N
DGTS(36:2)	764.6404	764.6413	1.2089	18:1/18:1	C46H86O7N
DGTS(38:5)	786.6248	786.6239	−1.1346	a	C48H84O7N
DGTS(40:9)	806.5935	806.5917	−2.2229	a	C50H80O7N
DGTS(40:8)	808.6091	808.6098	0.8873	a	C50H82O7N
**MGTS identified as [M+H]^+^**					
***MGTS(16:1)***	***472.3638***	***472.3631***	***−1.6157***	***16:1***	***C26H50O6N***
***MGTS(16:0)***	***474.3795***	***474.3795***	***−0.0942***	***16:0***	***C26H52O6N***
***MGTS(18:4)***	***494.3482***	***494.3486***	***0.9760***	***18:4***	***C28H48O6N***
MGTS(18:3)	496.3638	496.364	0.2825	18:3	C28H50O6N
MGTS(18:2)	498.3795	498.3794	−0.1732	a	C28H52O6N
MGTS(18:1)	500.3951	500.3961	1.9549	a	C28H54O6N

**Table 4:** *Grateloupia turuturu* phospholipids species identified by HILIC–LC–MS and MS/MS: type of adduct, lipid identity, calculated and observed mass, error, fatty acyl composition confirmed by MS/MS and formula. PC and LPC were identified as [M + H]^+^ ions, while PE, LPE, PG, LPG, PA, LPA, PI and LPI, PI-Cer were identified as [M – H]^−^ ions. The most abundant species are indicated in italics and in bold. C represents total carbon atoms and N represents the double bonds of fatty acid substituents. Fatty acyl chains were identified by MS/MS (a, assignments by mass accuracy, but without MS/MS; b, assignments by mass accuracy, MS/MS with diagnostic ions confirming the lipid class, but without information on fatty acyl chains). The representation C:N/C:N signifies the attribution of fatty acyl to the position sn-1/sn-2 according to the nomenclature of LIPIDMAPS (www.lipidmaps.org, accessed on 25 April 2021).

Lipid Species (C:N)	Calculated *m*/*z*	Observed *m*/*z*	Error (ppm)	Fatty Acyl Chains (C:N)	Formula
**PC identified as [M+H]^+^**					
PC(30:3)	700.4917	700.4902	−2.1598	a	C38H71NO8P
PC(30:0)	706.5387	706.5383	−0.506	b	C38H77NO8P
PC(32:4)	726.5074	726.5092	2.4576	a	C40H73NO8P
PC(32:3)	728.5230	728.524	1.3945	a	C40H75NO8P
PC(32:1)	732.5543	732.5519	−3.3249	16:0/16:1; 14:0/18:1	C40H79NO8P
PC(32:0)	734.5700	734.5701	0.1221	a	C40H81NO8P
PC(34:5)	752.5230	752.5205	−3.3016	14:0/20:5	C42H75NO8P
PC(34:4)	754.5387	754.5394	0.9047	14:0/20:4	C42H77NO8P
PC(34:3)	756.5543	756.5535	−1.0593	16:0/18:3	C42H79NO8P
PC(34:2)	758.5700	758.5674	−3.4063	16:0/18:2	C42H81NO8P
PC(34:1)	760.5856	760.5849	−0.9624	16:0/18:1	C42H83NO8P
PC(36:8)	774.5074	774.5039	−4.519	a	C44H73NO8P
PC(36:7)	776.5230	776.5222	−1.038	b	C44H75NO8P
PC(36:6)	778.5387	778.5372	−1.9005	20:5/16:1; 18:3/18:3	C44H77NO8P
PC(36:5)	780.5543	780.554	−0.4112	16:0/20:5; 18:3/18:2	C44H79NO8P
PC(36:4)	782.5700	782.5697	−0.342	16:0/20:4; 18:2/18:2	C44H81NO8P
PC(36:3)	784.5856	784.5854	−0.2952	b	C44H83NO8P
PC(36:2)	786.6013	786.6008	−0.5978	18:1/18:1	C44H85NO8P
PC(38:9)	800.5230	800.5217	−1.674	b	C46H75NO8P
PC(38:8)	802.5387	802.5376	−1.2955	18:3/20:5	C46H77NO8P
PC(38:7)	804.5543	804.5540	−0.4369	18:3/20:4	C46H79NO8P
PC(38:6)	806.5700	806.5695	−0.627	18:1/20:5	C46H81NO8P
PC(38:5)	808.5856	808.5850	−0.7921	18:1/20:4	C46H83NO8P
PC(40:10)	826.5387	826.5389	0.261	20:5/20:5	C48H77NO8P
***PC(40:9)***	***828.5543***	***828.5542***	***−0.2106***	***20:4/20:5***	***C48H79NO8P***
PC(40:8)	830.5700	830.5672	−3.4091	20:4/20:4	C48H81NO8P
PC(40:7)	832.5856	832.5820	−4.4128	b	C48H83NO8P
PC(42:11)	852.5543	852.5510	−3.9274	a	C50H79NO8P
**LPC identified as [M+H]^+^**					
LPC(14:0)	468.3090	468.3093	0.5844	14:0	C22H47NO7P
LPC(16:1)	494.3247	494.3235	−2.3082	16:1	C24H49NO7P
LPC(16:0)	496.3403	496.3400	−0.7179	16:0	C24H51NO7P
LPC(18:4)	516.3090	516.3074	−3.2093	a	C26H47NO7P
LPC(18:3)	518.3247	518.3232	−2.843	18:3	C26H49NO7P
LPC(18:2)	520.3403	520.3407	0.7228	18:2	C26H51NO7P
LPC(18:1)	522.3560	522.3563	0.5418	18:1	C26H53NO7P
LPC(20:5)	542.3247	542.3244	−0.5427	20:5	C28H49NO7P
***LPC(20:4)***	***544.3403***	***544.3406***	***0.4892***	***20:4***	***C28H51NO7P***
LPC(20:3)	546.3560	546.3540	−3.5931	20:3	C28H53NO7P
LPC(20:1)	550.3873	550.3881	1.5545	a	C28H57NO7P
LPC(22:6)	568.3403	568.3397	−1.1639	a	C30H51NO7P
LPC(22:5)	570.3560	570.3548	−2.1309	a	C30H53NO7P
**PE identified as [M − H]^−^**					
PE(30:2)	658.4448	658.4464	2.4664	a	C35H65NO8P
PE(30:1)	660.4604	660.4620	2.4129	16:1/14:0	C35H67NO8P
PE(30:0)	662.4761	662.4762	0.0882	a	C35H69NO8P
PE(32:3)	684.4604	684.4622	2.6111	a	C37H67NO8P
PE(32:2)	686.4761	686.4774	1.9274	16:1/16:1	C37H69O8NP
***PE(32:1)***	***688.4917***	***688.4932***	***2.1531***	***16:0/16:1***	***C37H71NO8P***
PE(34:3)	712.4917	712.4934	2.3376	16:0/18:3	C39H71O8NP
PE(34:2)	714.5074	714.5087	1.8928	16:1/18:1;16:0/18:2	C39H73NO8P
PE(34:1)	716.5230	716.5231	0.1676	16:0/18:1	C39H75NO8P
PE(36:5)	736.4917	736.4928	1.4659	16:0/20:5	C41H71O8NP
PE(36:2)	742.5387	742.5422	4.6648	18:1/18:1	C41H77O8NP
PE(38:8)	758.4760	758.4780	2.641	20:5/18:3	C43H69NO8P
PE(38:7)	760.4920	760.4931	1.4905	20:5/18:2; 20:4/18:3	C43H71NO8P
PE(38:5)	764.5230	764.5230	−0.0385	a	C43H75NO8P
**LPE identified as [M − H]^−^**					
LPE(14:0)	424.2464	424.2479	3.6359	a	C19H39NO7P
LPE(16:1)	450.2621	450.2634	2.9407	16:1	C21H41NO7P
***LPE(16:0)***	***452.2777***	***452.2787***	***2.2511***	***16:0***	***C21H43NO7P***
LPE(18:3)	474.2621	474.2631	2.3415	18:3	C23H41NO7P
LPE(18:2)	476.2777	476.2790	2.811	18:2	C23H43NO7P
LPE(18:1)	478.2934	478.2947	2.7404	18:1	C23H45NO7P
LPE(18:0)	480.3090	480.3109	3.961	a	C23H47NO7P
LPE(20:5)	498.2621	498.2634	2.6089	a	C25H41NO7P
LPE(20:4)	500.2777	500.2793	3.2658	20:4	C25H43NO7P
**PG identified as [M − H]^−^**					
PG(30:1)	691.4550	691.4562	1.7284	16:1/14:0	C36H68O10P
PG(30:0)	693.4707	693.4725	2.5772	a	C36H70O10P
PG(32:2)	717.4707	717.4725	2.4841	16:1/16:1; 18:2/14:0	C38H70O10P
PG(32:1)	719.4863	719.4864	0.2257	16:0/16:1	C38H72O10P
PG(32:0)	721.5020	721.5036	2.2107	a	C38H74O10P
PG(34:4)	741.4707	741.4706	−0.1774	20:4/14:0; 16:1/18:3	C40H70O10P
PG(34:3)	743.4863	743.4854	−1.1754	18:3/16:0	C40H72O10P
PG(34:2)	745.5020	745.5046	3.4986	18:1/16:1	C40H74O10P
PG(34:1)	747.5176	747.5189	1.6913	18:1/16:0	C40H76O10P
***PG(36:5)***	***767.4863***	***767.4878***	***1.9572***	***20:5/16:0; 20:4/16:1***	***C42H72O10P***
PG(36:5)OH	783.4829	783.4833	0.4648	20:5/16:0−OH	C42H72O10PO
PG(36:4)	769.5020	769.5039	2.4166	20:4/ 16:0; 18:2/18:2	C42H74O10P
PG(36:3)	771.5176	771.5181	0.6347	18:2/18:1	C42H76O10P
PG(36:2)	773.5333	773.5346	1.6895	18:1/18:1	C42H78O10P
PG(42:9)	843.5176	843.5169	−0.8453	a	C48H76O10P
**LPG identified as [M − H]^−^**					
LPG(14:0)	455.2410	455.2421	2.5057	14:0	C20H40O9P
LPG(16:1)	481.2566	481.2576	2.0266	16:1	C22H42O9P
LPG(16:0)	483.2723	483.2734	2.2252	16:0	C22H44O9P
LPG(16:0)OH	499.2677	499.2678	0.0841	16:0−OH	C22H44O10P
LPG(18:2)	507.2723	507.2740	3.1604	a	C24H44O9P
LPG(18:1)	509.2879	509.2888	1.649	18:1	C24H46O9P
***LPG(20:5)***	***529.2566***	***529.2577***	***1.9811***	***20:5***	***C26H42O9P***
LPG(20:4)	531.2723	531.2744	4.0749	20:4	C26H44O9P
LPG(22:5)	557.2879	557.2877	−0.3698	a	C28H46O9P
**PA identified as [M − H]^−^**					
PA(36:7)	689.4182	689.4178	−0.6446	a	C39H62O8P
PA(36:6)	691.4339	691.4359	2.941	16:1/20:5	C39H64O8P
PA(36:5)	693.4495	693.4508	1.8144	16:0/20:5	C39H66O8P
PA(40:10)	739.4339	739.4356	2.3648	a	C43H64O8P
PA(40:9)	741.4495	741.4496	0.0455	20:5/20:4	C43H66O8P
***PA(40:8)***	***743.4652***	***743.4660***	***1.1816***	***20:4/20:4***	***C43H68O8P***
**LPA identified as [M − H]^−^**					
LPA(20:4)	457.2355	457.2362	1.5001	a	C23H38O7P
**PI identified as [M − H]^−^**					
***PI(34:3)***	***831.5024***	***831.5030***	***0.7891***	***a***	***C43H76O13P***
PI(34:1)	835.5337	835.5307	−3.5756	16:0/18:1	C43H80O13P
PI(38:5)	883.5337	883.5337	0.0086	a	C47H80O13P
PI(40:10)	901.4867	901.4870	0.2814	a	C49H74O13P
PI(42:0)	949.6745	949.6721	−2.567	a	C51H98O13P
**LPI identified as [M − H]^−^**					
LPI(16:0)	571.2883	571.2897	2.3400	16:0	C25H48O12P
**PI-Cer identified as [M − H]^−^**					
PI−Cer(t38:0h)	868.5915	868.5901	−1.6315	a	C44H87NO13P
PI−Cer(t40:1h)	894.6077	894.6082	0.6170	b	C46H89NO13P
PI−Cer(t40:0h)	896.6228	896.6218	−1.0707	a	C46H91NO13P
PI−Cer(t42:0h)	924.6541	924.6509	−3.5109	a	C48H95NO13P
PI−Cer(t42:1h)	922.6390	922.6378	−1.2553	b	C48H93NO13P
***PI−Cer(t42:2h)***	***920.6233***	***920.6240***	***0.8190***	***t18:1/h24:1***	***C48H91NO13P***
PI−Cer(t43:2h)	934.6390	934.6403	1.3860	b	C49H93NO13P
PI−Cer(t44:2h)	948.6546	948.6553	0.7034	b	C50H95NO13P

**Table 5:** Fatty acid profile of lipid extracts of *Grateloupia turuturu*, expressed in µg mg^−1^ extract, µg g^−1^ of biomass DW and relative percentage (%, *w*/*w*) of mean ± SD, *n* = 3. The nutritional, health and quality parameters of the lipid extract were calculated using the fatty acid composition. Abbreviations: saturated fatty acids (SFA), monounsaturated fatty acids (MUFA), polyunsaturated fatty acids (PUFA), PUFA/SFA, PUFA *n*-6/*n*-3 and nutritive value (NVI) indices, health lipid indices, such as atherogenic (AI), thrombogenic (TI), hypocholesterolemic/hypercholesterolemic (h/H) and peroxidizability index (PI) ratios.

Fatty Acid	Mean ± SD Mean		
	(µg mg^−1^ Extract)	(µg g^−1^ DW Biomass)	(Relative Percentage, %)
C14:0	5.48 ± 0.75	57.11 ± 1.37	2.00 ± 0.07
C15:0	4.62 ± 0.21	48.41 ± 3.73	1.70 ± 0.13
C16:0	60.48 ± 10.44	628.89 ± 47.70	22.03 ± 0.98
C16:1*n*-9	4.28 ± 0.21	44.89 ± 3.43	1.58 ± 0.14
C16:1*n*-7	6.7 ± 0.50	70.12 ± 4.23	2.46 ± 0.12
C16:2*n*-6	4.37 ± 0.13	45.94 ± 4.81	1.61 ± 0.15
C17:0	3.78 ± 0.02	39.74 ± 4.75	1.39 ± 0.16
C17:1	4.83 ± 0.25	50.64 ± 3.88	1.78 ± 0.12
C18:0	15.02 ± 3.77	155.51 ± 23.29	5.44 ± 0.66
C18:1*n*-9	20.3 ± 1.94	212.35 ± 13.59	7.44 ± 0.24
C18:1*n*-7	11.2 ± 0.71	117.36 ± 8.03	4.12 ± 0.24
C18:2*n*-6	10.85 ± 0.78	113.8 ± 11.22	3.99 ± 0.28
C18:3*n*-6	5.79 ± 0.20	60.79 ± 5.51	2.13 ± 0.18
C18:3*n*-3	5.15 ± 0.07	54.12 ± 6.05	1.90 ± 0.22
C18:4*n*-3	5.14 ± 0.07	54.07 ± 5.92	1.90 ± 0.20
C20:0	6.22 ± 0.04	65.46 ± 8.23	2.30 ± 0.28
C20:3*n*-6	6.73 ± 0.29	70.58 ± 5.86	2.48 ± 0.19
C20:4*n*-6	35.41 ± 5.76	368.19 ± 20.56	12.91 ± 0.56
C20:5*n*-3	57.18 ± 8.74	595.19 ± 32.17	20.86 ± 0.56
**Sum**	**273.52 ± 34.49**	**2853.16 ± 139.33**	
SFA	95.6 ± 15.09	995.12 ± 66.55	37.49 ± 5.91
MUFA	42.48 ± 3.28	444.72 ± 27.65	16.66 ± 1.29
PUFA	126.24 ± 15.77	1316.74 ± 57.07	49.51 ± 6.18
*Σ* PUFA/*Σ* SFA	1.32 ± 0.04		
*Σ n*-6	58.77 ± 6.93	613.35 ± 27.28	
*Σ n*-3	67.47 ± 8.84	703.39 ± 30.47	
*n*-6/*n*-3	0.87 ± 0.01		
NVI	0.77 ± 0.03		
EPA/AA	1.62 ± 0.02		
AI	0.49 ± 0.02		
TI	0.32 ± 0.02		
h/H	2.4 ± 0.12		
PI, %	218.42 ± 30.47		

## Supplementary Materials

The following are available online at https://www.mdpi.com/article/10.3390/md19080414/s1, Table S1: Typical fragmentation patterns observed by Tandem Mass Spectrometry used to identify the head group and fatty acyl composition of polar lipids structures from *Grateloupia turuturu* (NL-neutral loss; PI-product ion), Figure S1: Representative examples of HILIC–LC–MS chromatograms of the polar lipids extracts from *G. turuturu* acquired on positive mode (a) and negative mode (b), Figure S2: LC–MS spectra of the galactolipids classes of *Grateloupia turuturu* monogalactosyl diacylglycerol, MGDG (a); monogalactosyl monoacylglycerol, MGMG (c); digalactosyl diacylglycerol, DGDG (e); and digalactosyl monoacylglycerol, DGMG (g), identified as [M + NH4]^+^ ions, Figure S3: LC–MS spectra of the sulfolipids classes of *Grateloupia turuturu* sulfoquinovosyl diacylglycerol, SQDG (a) and sulfoquinovosyl monoacylglycerol SQMG (c) identified as [M − H]^−^ ions, Figure S4: LC–MS of betaine lipids of *Grateloupia turuturu* diacyglyceryl-O-4′-(N,N,N-trimethyl) homoserine DGTS (a) and monoacyglyceryl-O-4′-(N,N,N-trimethyl) homoserine, MGTS (c), identified as [M + H]^+^ ions, Figure S5: LC–MS of the phospholipids classes of *Grateloupia turuturu* phosphatidylcholine, PC (a) and lyso-phosphatidylcholine, LPC (d) identified as [M + H]^+^ ions, Figure S6: LC–MS of the phospholipids classes phosphatidylethanolamine, PE (a) and lyso- phosphatidylethanolamine, LPE (d), identified as [M − H]^−^ ions, Figure S7: LC–MS of the phospholipids classes of *Grateloupia turuturu* phosphatidylglycerol, PG (a) and lyso- phosphatidylglycerol, LPG (c), identified as [M − H]^−^ ions, Figure S8: LC–MS of the phospholipid class of *Grateloupia turuturu* phosphatidic acid, PA (a), identified as [M − H]^−^ ion, Figure S9: LC–HCD–MS/MS of the selected [M − H]^−^ ion at *m*/*z* 835.53 of the phosphatidylinositol, Figure S10: LC–HCD–MS of phosphoinositol ceramide class of *Grateloupia turuturu* PI-Cer (a), identified as [M − H]^−^ ions, Figure S11: GC–MS chromatogram of the esterified fatty acid (FA) of *Grateloupia turuturu* lipid extract.
