# Supplementary material for: Polar Lipids Composition, Antioxidant and Anti-Inflammatory Activities of the Atlantic Red Seaweed Grateloupia turuturu
Source: Mar Drugs. 2021 Jul 26;19(8):414. doi: 10.3390/md19080414 (PMC8401436; doi:10.3390/md19080414)
Supplement: Supplementary file 1 [file marinedrugs-19-00414-s001.zip › marinedrugs-1286674-supplementary.pdf]

Supplementary Material

Polar lipids composition, antioxidant and anti-inflammatory activities of the Atlantic red seaweed *Grateloupia turuturu*

Elisabete da Costa [1,2,*], Tânia Melo [1,2], Mariana Reis [1,3], Pedro Domingues [1], Ricardo Calado [4], Maria Helena Abreu [3] and Maria Rosário Domingues [1,2]

[1] Mass Spectrometry Centre, LAQV-REQUIMTE, Department of Chemistry, University of Aveiro, Santiago University Campus, 3810-193 Aveiro, Portugal; taniamelo@ua.pt (T.M.); marianapreis@ua.pt (M.R.); p.domingues@ua.pt (P.D.); mrd@ua.pt (M.R.D.)

[2] CESAM-Centre for Environmental and Marine Studies, Department of Chemistry, University of Aveiro, Santiago University Campus 3810-193 Aveiro, Portugal

[3] ALGAplus—Production and Trading of Seaweed and Derived Products Lda., 3830-196 Ilhavo, Portugal; helena.abreu@algaplus.pt (M.H.A.)

[4] ECOMARE, CESAM-Centre for Environmental and Marine Studies Department of Biology, University of Aveiro, Santiago University Campus, 3810-193 Aveiro, Portugal; rjcalado@ua.pt (R.C.) * Correspondence: elisabetecosta@ua.pt; Tel.: +351-234-370-696

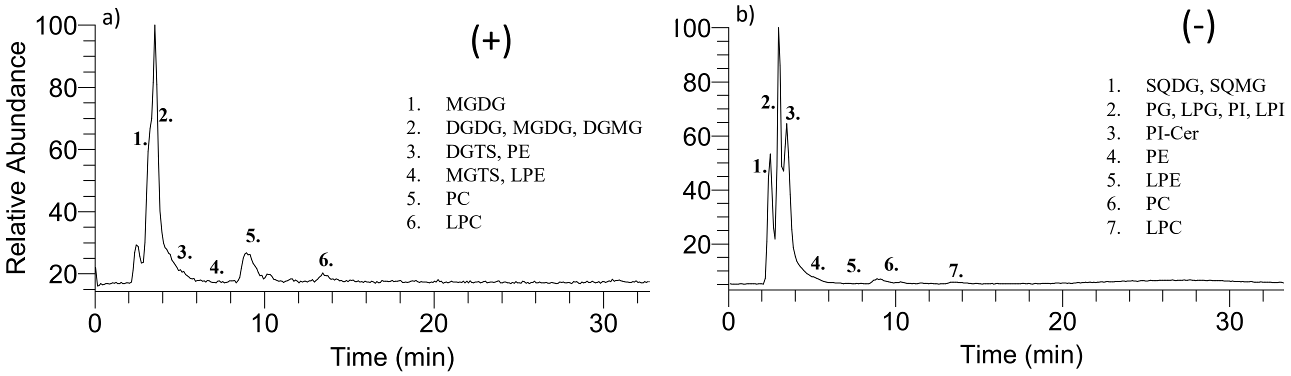

Figure S1. Representative examples of HILIC–LC–MS chromatograms of the polar lipids extracts from *Grateloupia turuturu* acquired on positive mode (a) and negative mode (b).

Table S1. Typical fragmentation patterns observed by Tandem Mass Spectrometry used to identify the head group and fatty acyl composition of polar lipids structures from *Grateloupia turuturu* (NL-neutral loss; PI-product ion)

**Lipid class/subclass**	**Ion mode**	**Precursor ion (MS)**	**Fragmentation patterns**	
			**Lipid class**	**Fatty acyl composition**
**Glycolipids**				
MGDG; MGMG	Positive	$[M + NH_4]^+$	Neutral loss (NL) of 197 Da	Product ion (PI) $[RCO + 74]^+$
DGDG; DGMG	Positive	$[M + NH_4]^+$	NL of 341 and 359 Da	PI $[RCO + 74]^+$; $[RCO]^+$
SQDG; SQMG	Negative	$[M - H]^-$	PI at *m/z* 225.0	NL of RCOOH
**Phospholipids**				
PG; LPG	Negative	$[M - H]^-$	PI at *m/z* 227.1 and *m/z* 153.0	PI $(RCOO^-)$
PC; LPC	Positive	$[M + H]^+$	PI at *m/z* 184.1	
	Negative	$[M + CH_3COO]^-$		PI $(RCOO^-)$
PE; LPE	Positive	$[M + H]^+$		NL of 141 Da
	Negative	$[M - H]^-$		PI $(RCOO^-)$
PI; LPI	Negative	$[M - H]^-$	PI at *m/z* 241.0 and *m/z* 153.0	PI (COO^-)<br>NL of $R_{1,2}COOH$
PA	Negative	$[M - H]^-$	PI at *m/z* 153.0	PI $(RCOO^-)$
**Inositephosphoceramides**				
PI-Cer	Positive	$[M + H]^+$	PI at *m/z* 262.2, *m/z* 280.3 and *m/z* 298.3	
	Negative	$[M - H]^-$	PI at *m/z* 241and *m/z* 259	NL of 180 Da
**Betaine lipids**				
DGTS; MGTS	Positive	$[M + H]^+$	PI at *m/z* 236.1 and *m/z* 144.1	NL of R=C=O, NL of RCOOH

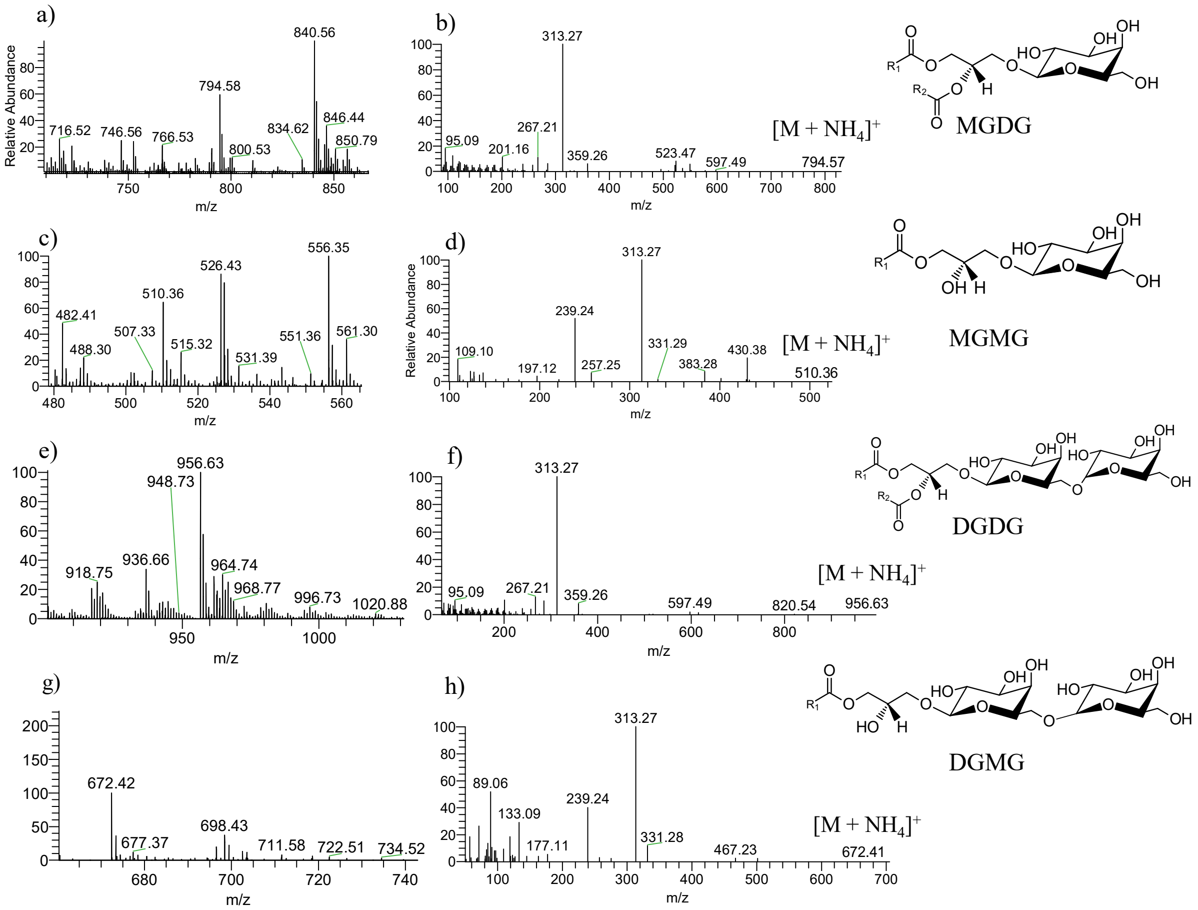

Figure S2. LC–MS spectra of the galactolipids classes of *Grateloupia turuturu* monogalactosyl diacylglycerol, MGDG (a); monogalactosyl monoacylglycerol, MGMG (c); digalactosyl diacylglycerol, DGDG (e); and digalactosyl monoacylglycerol, DGMG (g), identified as $[M + NH_4]^+$ ions. HCD–MS/MS of the selected ions seen at *m/z* 794.57 (b) identified as MGDG (36:5), at *m/z* 510.36 (d) identified as MGMG (16:0), at *m/z* 956.63 (f) identified as DGDG (36:5), and at *m/z* 672.41 (h), identified as DGMG (16:0), are shown. The main fragmentation pathway of MGDG and MGMG is the neutral loss of the galactosyl polar head group combined loss of NH_3 plus loss of hexose unit (−197 Da) with the formation of the product ion seen at *m/z* 597.49 (MGDG, (b)) and 313.27 (MGMG, (d)). The main fragmentation pathway of DGDG and DGMG is the neutral loss of the digalactosyl polar head group combined with loss of NH_3 (−359 Da) with formation of the product ion at *m/z* 597.49 (DGDG, (f)) and 313.27 (DGMG, (h)). The fatty acyl substituents were identified by the acylium product ions plus 74 Da $[RCO + 74]^+$, seen at *m/z* 313.27 and *m/z* 359.26, that corresponded to the fatty acyl chains 16:0 and 20:5, as observed in Figures (b) and (f) for MGDG (20:5_16:0) and DGDG(20:5_16:0), respectively. The acylium product ion plus 74 Da $[RCO + 74]^+$ at *m/z* 313.27 and the acylium product ion seen at *m/z* 239.24 $[RCO]^+$ corresponded to the fatty acyl group 16:0 that allows to confirm the presence of the d) MGMG (16:0) and h) DGMG (16:0).

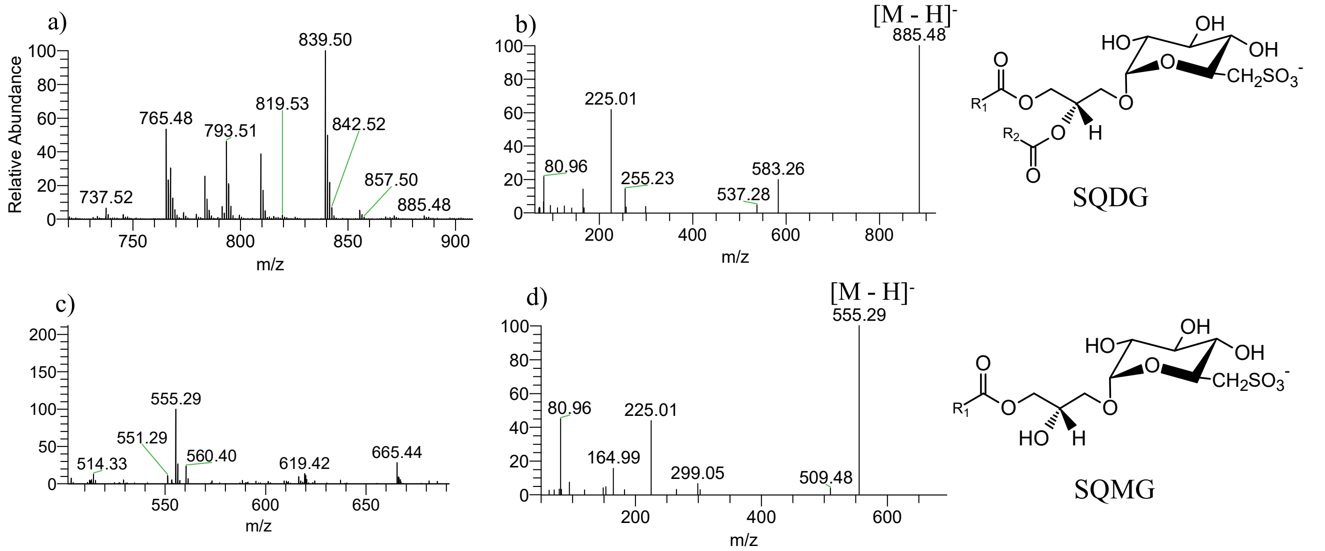

Figure S3. LC–MS spectra of the sulfolipids classes of *Grateloupia turuturu* sulfoquinovosyl diacylglycerol, SQDG (a), and sulfoquinovosyl monoacylglycerol SQMG (c) identified as $[M - H]^-$. HCD–MS/MS spectra of selected ions seen at *m/z* 885.48 (b) identified as (40:10) and *m/z* 555.29 (d) identified as (16:0) are depicted. The diagnostic product ion of these classes is the ion at *m/z* 225.01 corresponding to the dehydrosulfoglycosyl anion $[C_6H_9O_7S]^-$ that allows to identify the polar head group. The typical NL of RCOOH, allows to confirm fatty acyl composition and can be seen by the product ion at *m/z* 583.26, in the Figure (b), results from the neutral loss of fatty acyl chain 20:5 (-RCOOH, – 302 Da) that allows the identification of SQDG (20:5/20:5), while the product ion at *m/z* 299.05 (Figure d) corresponds to neutral loss of fatty acyl group 16:0 (-RCOOH, –256 Da) obtained from SQMG (16:0).

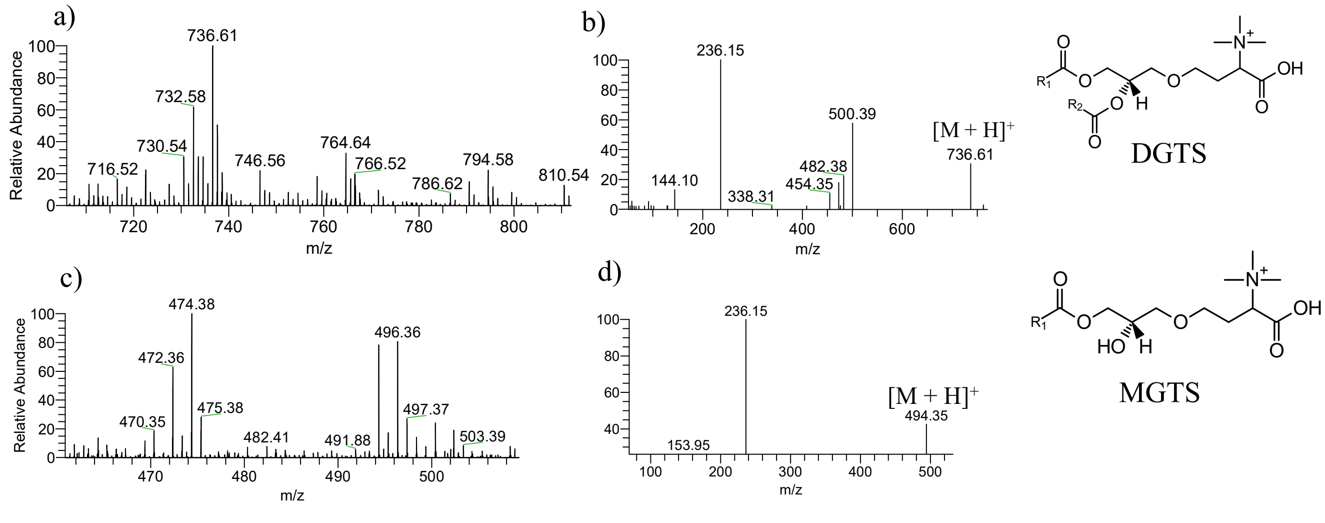

Figure S4. LC–MS of betaine lipids of *Grateloupia turuturu* diacyglyceryl-O-4′-(N,N,N-trimethyl) homoserine DGTS (a), and monoacyglyceryl-O-4′-(N,N,N-trimethyl) homoserine, MGTS (c), identified as $[M + H]^+$ ions. HCD–MS/MS spectra of selected ions seen at *m/z* 736.61 (b) identified as DGTS (34:2), and at *m/z* 494.35 identified as MGTS (18:4) (d) are depicted. The diagnostic product ion of these classes is the ion at *m/z* 236.15 that resulted from the loss of fatty acyl group as ketenes and allow to identify the polar head group, as depicted in (b) and (d). The typical NL of free carboxylic acid RCOOH and ketene form R=C=O allows to confirm fatty acyl composition and can be seen by the product ion at *m/z* 482.38 and *m/z* 500.39 (b), corresponding to the neutral loss of 16:1 fatty acid, and the product ion at *m/z* 454.35 corresponding to the neutral loss of the free carboxylic acid RCOOH of the fatty acid 18:1, that allows the identification of DGTS (18:1_16:1). In the Figure S4 (d), the product ion at *m/z* 236.15, corresponding to the neutral loss of the ketene form R=C=O of the 18:4, assigns the fatty acyl chain composition MGTS (18:4).

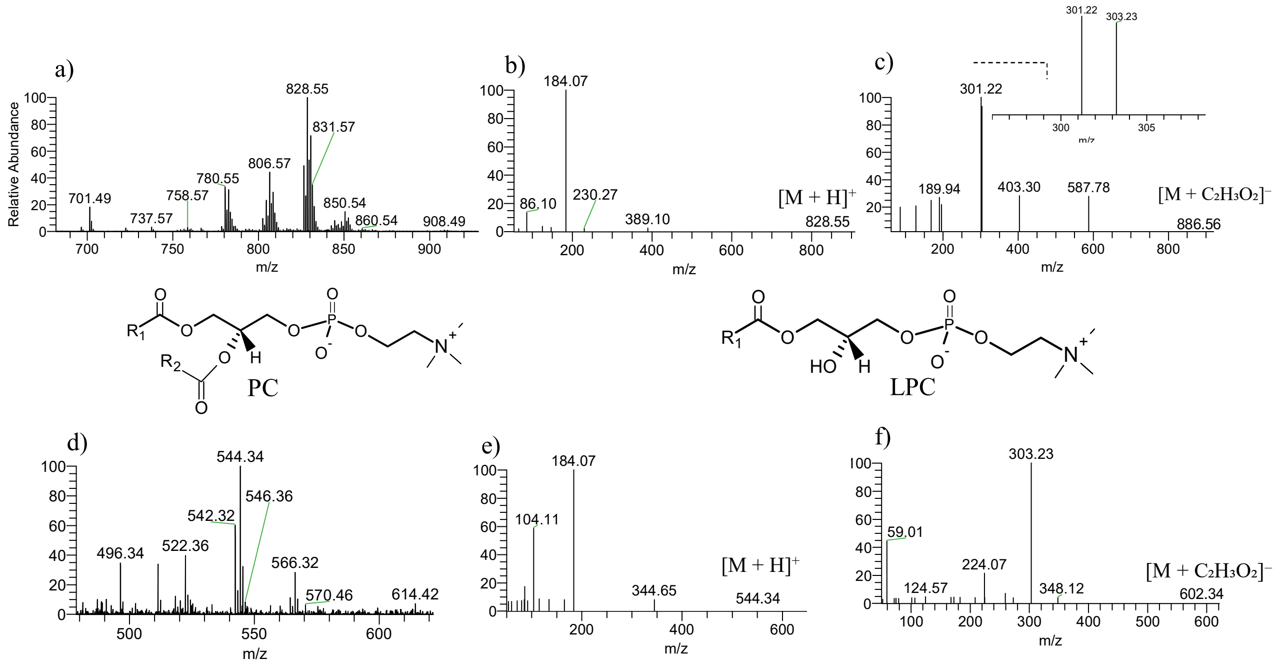

Figure S5. LC–MS of the phospholipids classes of *Grateloupia turuturu* phosphatidylcholine, PC (a), and lyso-phosphatidylcholine, LPC (d) identified as $[M + H]^+$ ions. HCD–MS/MS spectra of selected ion seen at *m/z* 828.55 (b), identified as PC (40:9), and at *m/z* 544.34 (e) identified as LPC (20:4) are depicted. The diagnostic product ion of these classes is the ion seen at *m/z* 184.07, corresponding to the phosphocholine anion $C_5H_{15}NO_4P^+$ that allows to identify the polar head group. The typical presence of carboxylate anions seen in the HCD–MS/MS spectra of the $[M + CH_3COO]^-$ ions allows to confirm fatty acyl composition, as demonstrated in the spectra of the *m/z* 886.56 (c) and *m/z* 602.34 (f) ions. The product ions seen at *m/z* 301.22 and *m/z* 303.23 (c) correspond to fatty acyl carboxylate anions 20:5 (R_2COO^-) and 20:4 (R_1COO^-) and allow to identify the fatty acyl composition of PC (20:4/20:5); and at the product ion seen at *m/z* 303.23 (f) corresponds to fatty acyl carboxylate anion ($RCOO^-$) and allow to identify the fatty acyl composition of LPC (20:4).

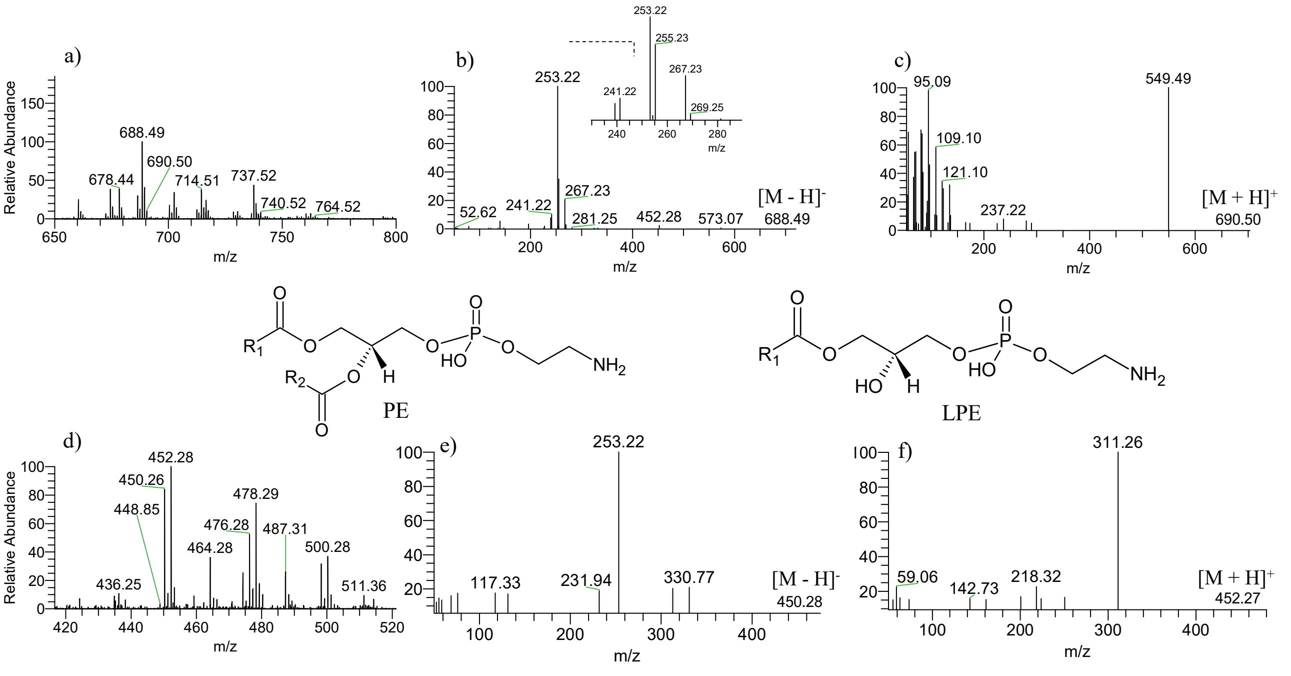

Figure S6. LC–MS of the phospholipids classes of *Grateloupia turuturu* phosphatidylethanolamine, PE (a), and lyso-phosphatidylethanolamine, LPE (d), identified as $[M - H]^-$ ions. HCD–MS/MS spectra of selected ions at *m/z* 688.49 (b) identified as PE (32:1), and *m/z* 450.28 (e) identified as LPE (16:1), are shown. In these spectra, the product ions seen at *m/z* 253.22 and 255.23 (b) correspond to fatty acyl carboxylate anion ($RCOO^-$) and allow to identify the fatty acyl composition 16:0 (R_1COO^-) and 16:1 (R_2COO^-) of PE (16:0/16:1); the product ions seen at *m/z* 253.22 correspond to fatty acyl carboxylate anion 16:1 ($RCOO^-$) and allows the identification of the fatty acyl composition of LPE (16:1) (e). The typical loss of 141 Da observed in the HCD–MS/MS spectra of $[M + H]^+$ ions of PE (c) and LPE (f) allows to identify the phosphoethanolamine head group.

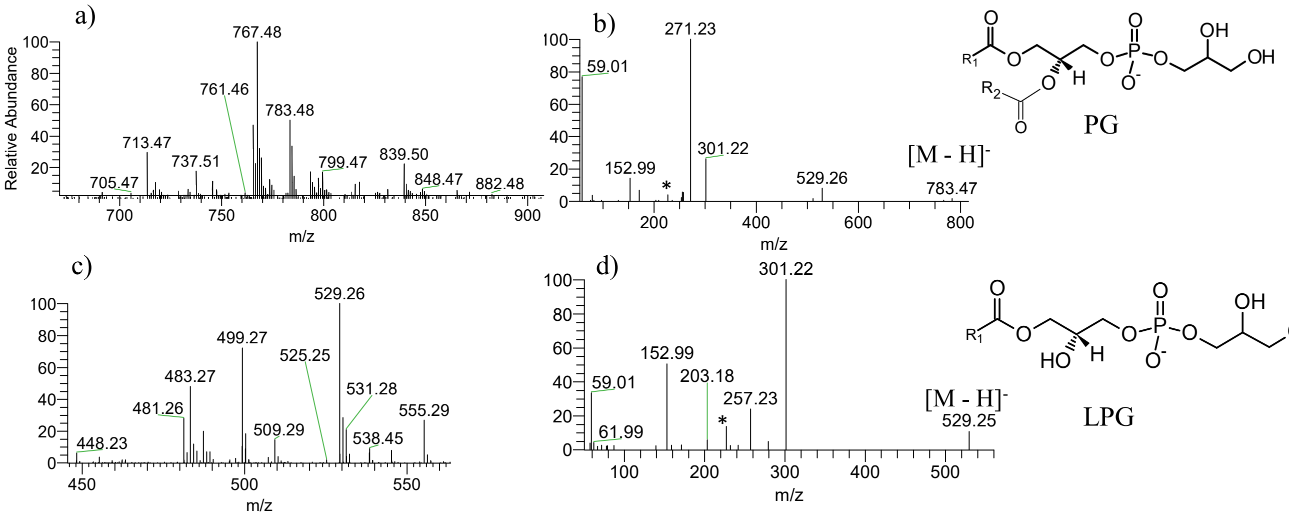

Figure S7. LC–MS of the phospholipids classes of *Grateloupia turuturu* phosphatidylglycerol, PG (a), and lyso-phosphatidylglycerol, LPG (c), identified as $[M - H]^-$ ions. HCD–MS/MS spectra of selected $[M - H]^-$ ions seen at *m/z* 783.47 (b) identified as PG (36:5)OH, and *m/z* 529.25 (d) identified as LPG (20:5) are shown. The diagnostic product ion of these classes is the ion seen at *m/z* 227.2 (combined loss of ketene, R=C=O, and the fatty acid, RCOOH,*) and 152.99 (phosphoglycerol derivative) that allow to identify the polar head group. The typical presence of carboxylate anions are seen at *m/z* 271.23 and *m/z* 301.22 (b) correspond to fatty acyl carboxylate anions 16:0-OH (R_2COO^-) and 20:5 (R_1COO^-), attributed to PG (20:5/16:0-OH); and the product ion seen at *m/z* 301.22 (d) correspond to fatty acyl carboxylate anion 20:5 ($RCOO^-$), attributed to LPG (20:5).

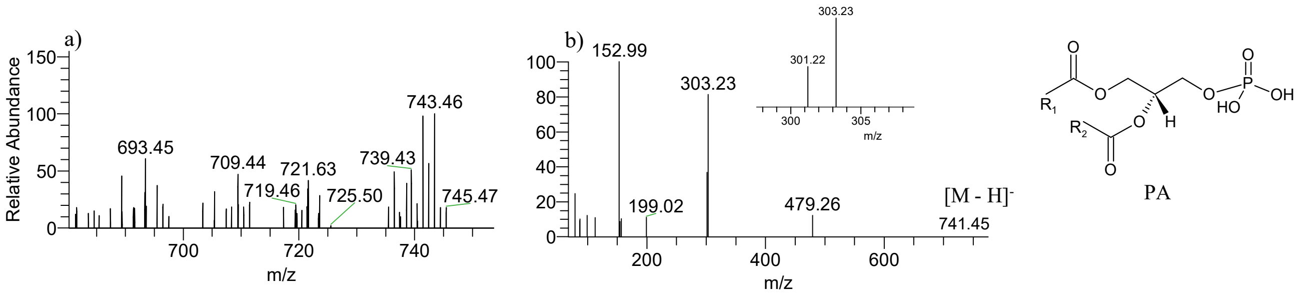

Figure S8. LC–MS of the phospholipid class of *Grateloupia turuturu* phosphatidic acid, PA (a) identified as [M − H]⁻ ion. HCD–MS/MS spectra of the selected ion seen at *m/z* 741.45 (b), identified as PA (40:9), is shown. The diagnostic product ion of this class is the ion seen at *m/z* 152.99 and attributed to cyclic phosphate anion. The typical presence of carboxylate anions seen at *m/z* 301.22 and *m/z* 303.23 assign the fatty acyl carboxylate anions 20:5 (R_1COO^-) and 20:4 (R_2COO^-) obtained from PA (20:5/20:4).

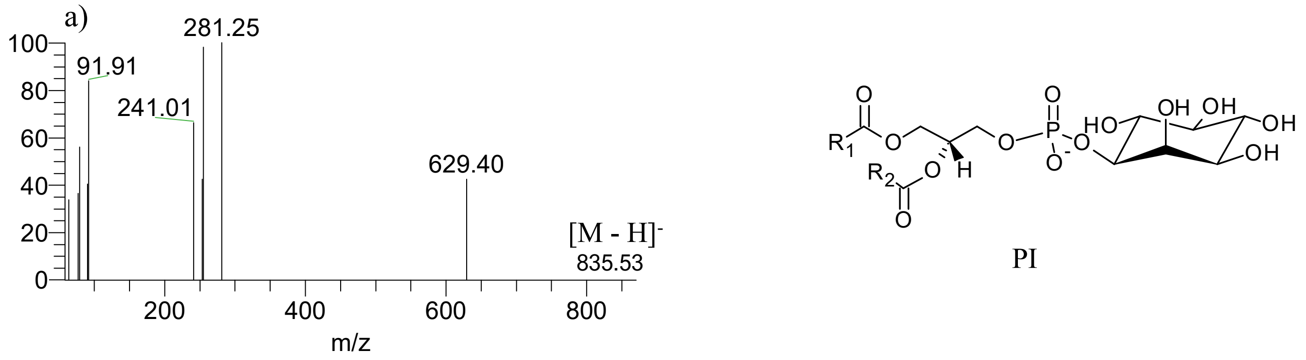

Figure S9. LC–HCD–MS/MS of the selected [M − H]⁻ ion at *m/z* 835.53 of the phosphatidylinositol lipid species PI (34:1) (a) of *Grateloupia turuturu* is shown. The diagnostic product ion of this class is the ion seen at *m/z* 241.01 corresponds to the cyclic anion of inositol phosphate from the polar head. The product ion at *m/z* 255.2 and *m/z* 281.25 (a), corresponding to fatty acyl carboxylate anions 16:0 (R_1COO^-) and 18:1 (R_2COO^-) allow to identify PI (16:0/18:1).

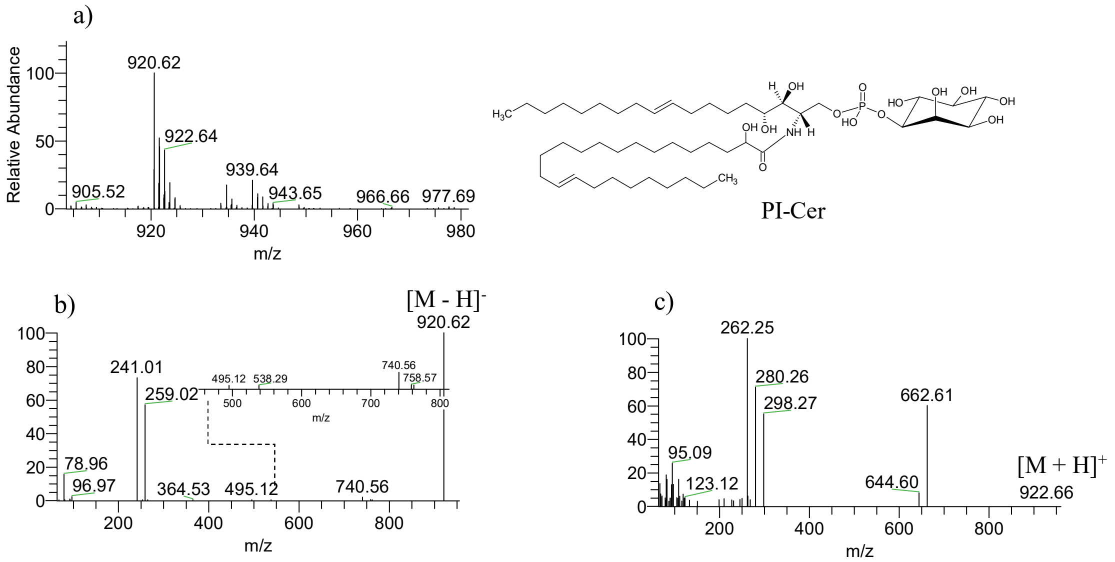

Figure S10. LC–HCD–MS of phosphoinositol ceramide class of *Grateloupia turuturu* PI-Cer (a), identified as [M − H]$^-$ ions. HCD–MS/MS spectra of the selected ion seen at *m/z* 920.62, identified as PI-Cer (t18:1/h24:1) is shown. The diagnostic product ion of this class are the ions seen at *m/z* 241.01, corresponding to an inositol-1,2-cyclic phosphate anion, and at *m/z* 259.02 corresponding to an inositol monophosphate anion. The ion at *m/z* 538.29 (− 382), corresponding to loss of a 24:1(OH)-fatty acyl substituent (b). The spectrum also contains the ions at *m/z* 740.56 (−180Da), arising from losses of an inositol residues. HCD–MS/MS of [M + H]$^+$ ion at *m/z* 922.66 (c) showed the sphingoid base fragment ion *t*18:1 at *m/z* 298.27, and the product ions at *m/z* 280.26 at *m/z* 262.25 allowed to complete the identification of the trihydroxy sphingoid bases due to the loss of one and two hydroxy groups, respectively.

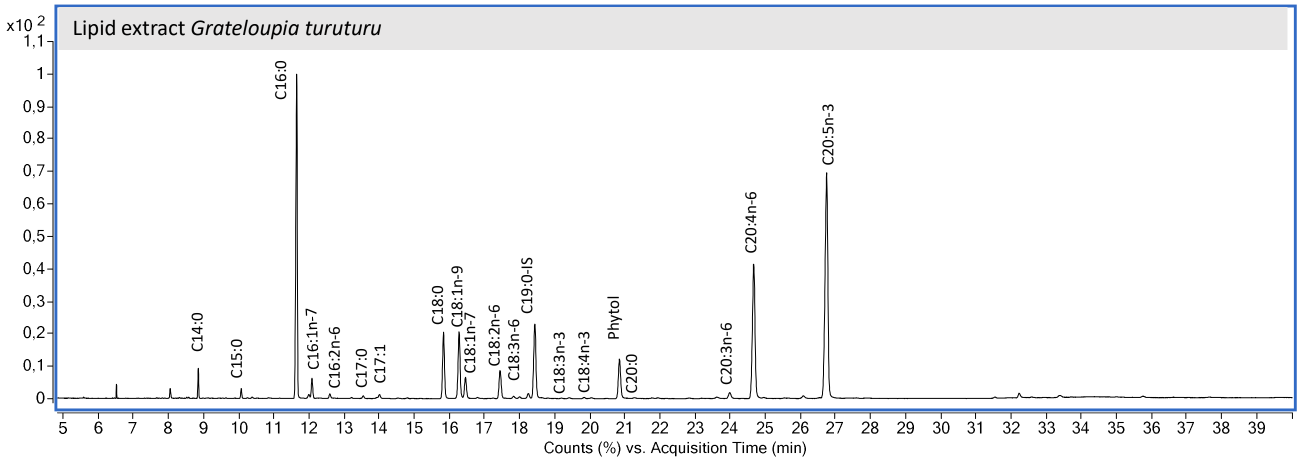

Figure S11. GC–MS chromatogram of the esterified fatty acids (FA) of *Grateloupia turuturu* lipid extract.
